# Cu(OTf)_2_‐Mediated Cross‐Coupling of Nitriles and N‐Heterocycles with Arylboronic Acids to Generate Nitrilium and Pyridinium Products**

**DOI:** 10.1002/anie.202016811

**Published:** 2021-02-26

**Authors:** Nicola L. Bell, Chao Xu, James W. B. Fyfe, Julien C. Vantourout, Jeremy Brals, Sonia Chabbra, Bela E. Bode, David B. Cordes, Alexandra M. Z. Slawin, Thomas M. McGuire, Allan J. B. Watson

**Affiliations:** ^1^ EaStCHEM School of Chemistry University of St Andrews North Haugh St Andrews Fife KY16 9ST UK; ^2^ GlaxoSmithKline Medicines Research Centre Gunnels Wood Road Stevenage Hertfordshire SG1 2NY UK; ^3^ AstraZeneca Darwin Building, Unit 310, Cambridge Science Park, Milton Road Cambridge CB4 0WG UK

**Keywords:** arylation, boron, copper, cross-coupling, reaction mechanisms

## Abstract

Metal‐catalyzed C–N cross‐coupling generally forms C−N bonds by reductive elimination from metal complexes bearing covalent C‐ and N‐ligands. We have identified a Cu‐mediated C–N cross‐coupling that uses a dative N‐ligand in the bond‐forming event, which, in contrast to conventional methods, generates reactive cationic products. Mechanistic studies suggest the process operates via transmetalation of an aryl organoboron to a Cu^II^ complex bearing neutral N‐ligands, such as nitriles or N‐heterocycles. Subsequent generation of a putative Cu^III^ complex enables the oxidative C–N coupling to take place, delivering nitrilium intermediates and pyridinium products. The reaction is general for a range of N(sp) and N(sp^2^) precursors and can be applied to drug synthesis and late‐stage N‐arylation, and the limitations in the methodology are mechanistically evidenced.

## Graphical abstract

Cu(OTf)_2_ promotes the oxidative arylation of nitriles and N‐heterocycles to generate ionic products. Mechanistic studies suggest the process operates via reductive elimination from a Cu^III^ complex bearing neutral N‐ligands to generate nitrilium and pyridinium products. The reaction is general for a range of N(sp) and N(sp^2^) precursors and can be applied to drug synthesis and late‐stage N‐arylation.

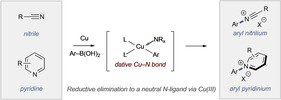

## Introduction

**Scheme 1:**
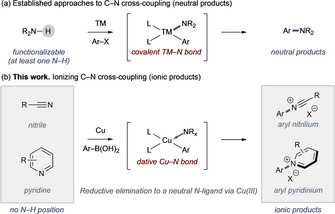
a) General approach to cross‐coupling. b) This work: cross‐coupling to unconventional substrates. Ar=aryl, TM=transition metal.

Transition metal‐mediated C‐N cross‐coupling is an essential synthetic method, used extensively throughout the chemical industry for the synthesis of pharmaceuticals, agrochemicals, natural products, and materials.[^1^, ^2^, ^3^, ^4^, ^5^, ^6^, ^7^, ^8^] The development of new or improved processes for C−N bond construction remains a continual inspiration for metal‐based reaction development. Despite a broad diversity and subtlety in the mechanism of these methods, the basic premise of the reaction involves a series of individual mechanistic steps, e.g., oxidative addition, transmetalation, and/or deprotonation, to allow access to a key metal complex bearing formally anionic, covalently bound C‐ and N‐ligands (Scheme 1 a). This complex undergoes reductive elimination to deliver a neutral product, which is produced regardless of whether the catalysis itself is electroneutral (e.g., the Buchwald‐Hartwig or Ullmann‐Goldberg reactions) or oxidative (e.g., the Chan‐Lam reaction).[^6^, ^7^, ^8^, ^9^]

In these processes, the N‐ligand originates from a precursor amine or amine‐derived substrate bearing at least one functionalizable N−H, which undergoes deprotonation at some stage in the reaction mechanism to deliver the required anionic N‐ligand. This limits the scope of established processes to substrates with at least one N−H.

However, it should be noted that the direct N‐arylation of substrates without a functionalizable site is known. N‐arylation of nitriles and N‐heterocycles has been achieved with or without transition metal activators, for example, using diaryliodonium salts.[^10^, ^11^] With Cu‐promoted processes,[^10^, ^11^] these are mechanistically ambiguous, with no evidence for a metal‐centered reductive elimination. These processes have also been rationalized as direct arylation using the increased electrophilicity of the aryl transfer reagent via Lewis acid activation.^[12]^ More specifically, while Cu^I^ has been shown to slightly accelerate aryl transfer with diaryliodoniums, these processes also proceed effectively without Cu^I^,[^13^, ^14^] consistent with observed general reactivity of this class of reagents^[15]^ and related reactive aryl transfer reagents, such as aryldiazonium salts.^[16]^

Here, we report the discovery, mechanistic rationale, example scope, and limitations of a Cu‐mediated C‐N cross‐coupling method that promotes reductive elimination to neutral N‐ligands, such as nitriles and N‐heterocycles generating reactive cationic products (Scheme 1 b).

## Results and Discussion

**Scheme 2:**
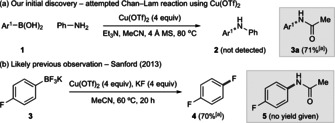
Observations of Cu‐mediated nitrile arylation. [a] Determined by HPLC. Ar^1^=*p*‐PhC_6_H_4_. Tf=trifluoromethylsulfonyl.

During investigations to rationalize the reactivity of Cu^II^ sources in standard Chan‐Lam reactions, the amide product **3 a** was identified in good yield when the reaction of arylboronic acid **1** with aniline was attempted with Cu(OTf)_2_ in MeCN as solvent (Scheme 2 a). A similar observation was made by Sanford during studies of fluorodeboronation, which also used stoichiometric Cu(OTf)_2_ (4 equiv) in MeCN (Scheme 2 b).^[17]^ We were intrigued by this observation since hydrolysis of MeCN to acetamide followed by Chan‐Lam‐type N‐arylation seemed unlikely—we have previously attempted Chan‐Lam arylations of amides using Cu(OTf)_2_ and found this to be problematic (vide infra). Consequently, we sought to understand the origin of this coupling process.

**Scheme 3:**
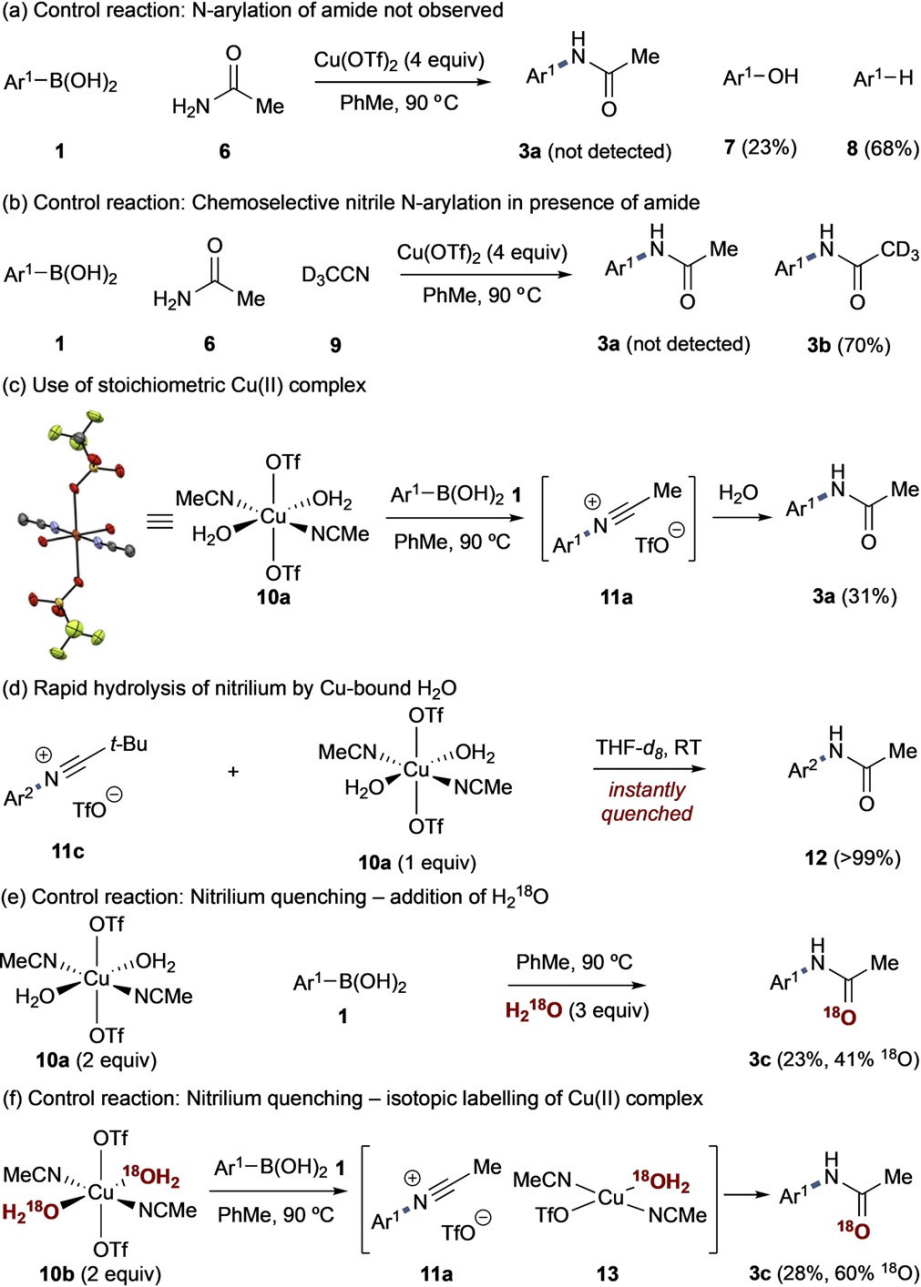
Control reactions. Ar^1^=*p*‐PhC_6_H_4_; Ar^2^=*p*‐(F_3_CO)C_6_H_4_.

Control experiments indicated the possibility of an alternative pathway. The Chan‐Lam arylation of acetamide **6** using Cu(OTf)_2_ in PhMe does not provide the N‐aryl product. Instead, the products of aryl‐boronic acid oxidation and protodeboronation were observed and represented the almost complete mass balance (Scheme 3 a)—protodeboronation was also noted as an issue in Sanford's study^[17]^ and is a common problem for Cu‐mediated reactions of organoborons.[^18^, ^19^] Separate experiments (Table S1) indicated that the same conditions did not lead to nitrile hydrolysis.^[20]^ The competition reaction of **1** with acetamide and D_3_CCN in the presence of Cu(OTf)_2_ led to the deuterated acetamide product **3 b** exclusively, further supporting the absence of a Chan‐Lam pathway and indicating selectivity for nitrile (Scheme 3 b).

To rationalize these initial observations, we considered a reaction pathway that proceeded via formation of a nitrilium intermediate formed by Cu‐mediated N‐arylation of the nitrile. N‐arylation of nitriles is known using highly reactive aryl transfer agents, such as iodonium and diazonium salts;[^10^, ^16^] however, oxidative coupling of nitriles with arylboronic acids is unknown. Accordingly, we sought to establish if an oxidative coupling pathway was operational.

Treatment of Cu(OTf)_2_ with H_2_O in MeCN leads to a stable and isolable complex Cu^II^(OTf)_2_(H_2_O)_2_(MeCN)_2_ (**10 a**, Scheme 3 c).^[21]^ Heating this complex with **1** lead to the observed acetamide **3 a**, which we propose proceeds through nitrilium **11 a**, suggesting possible formation and involvement of **10 a** in the reaction.^[22]^

Nitrilium ions are highly reactive electrophiles capable of a variety of bond forming processes with nucleophiles;^[23]^ however, extensive experimentation to intercept the proposed nitrilium **11 a** were unsuccessful and afforded a mixture of amide and returned starting material (Tables S2 and S3). We therefore attributed amide formation to hydrolysis of the nitrilium with H_2_O present in the reaction mixture, arising either from boroxine formation from **1** or Cu‐bound H_2_O in **10 a**—H_2_O could not be excluded in preparation of stoichiometric Cu(OTf)_2_ nitrile complexes as noted above.

Independent preparation of stable nitrilium **11 c** ^[24]^ and treatment with **10 a** led to instantaneous hydrolysis, highlighting the lability of Cu‐bound H_2_O (Scheme 3 d). To probe the origin of H_2_O in the acetamide product, we undertook labelling experiments. Addition of H_2_^18^O to the reaction of **10 a** with **1** led to 41 % ^18^O incorporation in the product **3 c**, consistent with the ^16^O:^18^O stoichiometry (Scheme 3 e). Preparation of ^18^O‐labelled complex **10 b** was successful; however, the ^18^O incorporation could not be quantified due to lability of the dative ligands. Indeed, despite obtaining crystal structure data of **10 a** and **10 b** (identical), HRMS analyses were uniformly unsuccessful. Use of **10 b** in the absence of additional H_2_O gave **3 c** in comparable yield to the reaction of **10 a** and with 60 % ^18^O incorporation (Scheme 3 f).

The inability to trap the nitrilium by any nucleophile other than H_2_O suggests that nitrilium quenching may be occurring from H_2_O in solution, a Cu aquo species (e.g., **10 a**/**10 b**), or from a Cu^I^ complex liberated after reductive elimination (e.g., **13**, Scheme 3 f).

**Scheme 4:**
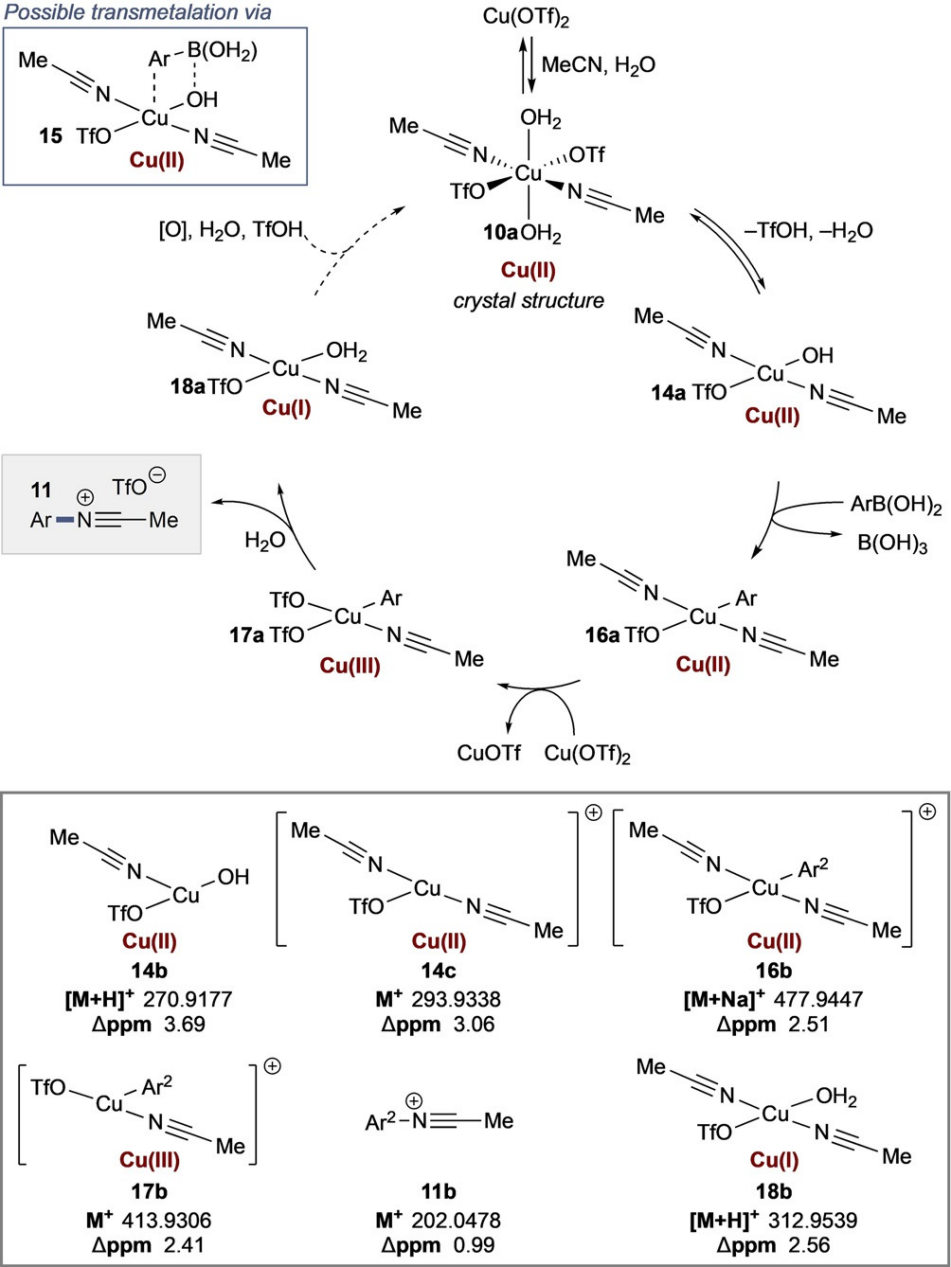
Proposed mechanism with observed HRMS mass ions.

To further substantiate this nitrilium proposal, HRMS analysis of reaction mixtures identified a series of mass ions that allowed the following mechanism to be proposed (Scheme 4).^[25]^

We propose that Cu(OTf)_2_ forms **10 a** (crystal structure obtained from reaction mixtures). Loss of TfOH and H_2_O gives **14 a**–mass ions consistent with [**14 a**–MeCN] (**14 b**) and [**14 a**–H_2_O] (**14 c**) were found. Transmetalation then gives **16 a** (**16 b** found), possibly via a pathway consistent to the Chan‐Lam amination (**15**).^[26]^ Disproportionation of **16 a** gives the key Cu^III^ intermediate **17 a** with [**17 a**–TfO^−^] (**17 b**) found,[^26^, ^27^, ^28^] allowing formation of the nitrilium product **11** (**11 b** found). Mass ions consistent with the proposed Cu^I^ aquo complex **18 a** were detected (**18 b**), consistent with the quenching proposal outlined in Scheme 3 f. Stoichiometric Cu(OTf)_2_ was exclusively effective—other Cu sources failed to promote the reaction (Table S7). Extensive investigation failed to allow this process to operate with catalytic Cu(OTf)_2_—the addition of terminal oxidants led to issues of organoboron oxidation and rendering Cu turnover (Scheme 4, dotted line) irrelevant (Table S9). The same turnover issues in systems using Cu(OTf)_2_ and CuOTf have been observed in C‐F bond formation by Sanford^[17]^ and Hartwig,^[29]^ respectively, where 3–4 equivalents of Cu were necessary for reaction efficiency. This problem remains unresolved for many Cu(OTf)_2_‐based processes.^[30]^

The proposed nitrilium ions were observable by HRMS; however, the inability to intercept the proposed nitrilium with other nucleophiles was unsatisfactory. Specifically, this invites further scrutiny of the proposed key C−N bond forming event in Scheme 4—the potential for an on‐metal hydrolysis cannot be excluded. We therefore sought to demonstrate the C−N bond forming process using a system that would allow unambiguous identification C−N bond formation produced from reductive elimination to a neutral N‐ligand on Cu^III^.

**Scheme 5:**
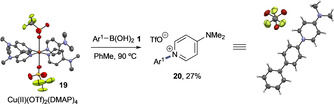
Cross‐coupling to N(sp^2^) via DMAP complex **19**. Ar^1^=*p*‐PhC_6_H_4_, DMAP=4‐dimethylaminopyridine.

Treatment of Cu(OTf)_2_ with DMAP allowed formation of Cu^II^(OTf)_2_(DMAP)_4_ **19** and its structure unambiguously confirmed by X‐ray (Scheme 5).

Complex **19** is similar to the nitrile complex **10 a**; however, this can be prepared without aquo ligands. Under the same reaction conditions used in Scheme 3 c, **19** leads to a similar C−N bond formation giving N‐aryl pyridinium **20** and in similar yield to the nitrile process. **20** was characterized unambiguously by spectroscopy and X‐ray, providing strong support for C‐N cross‐coupling via Cu^III^. We propose this reaction to follow a similar course to that proposed in Scheme 4. Single electron pathways via oxidation of DMAP by Cu^II^ were proposed to be unlikely based on oxidation potentials and EPR analysis (vide infra).[^33^, ^34^, ^35^]

**Scheme 6:**
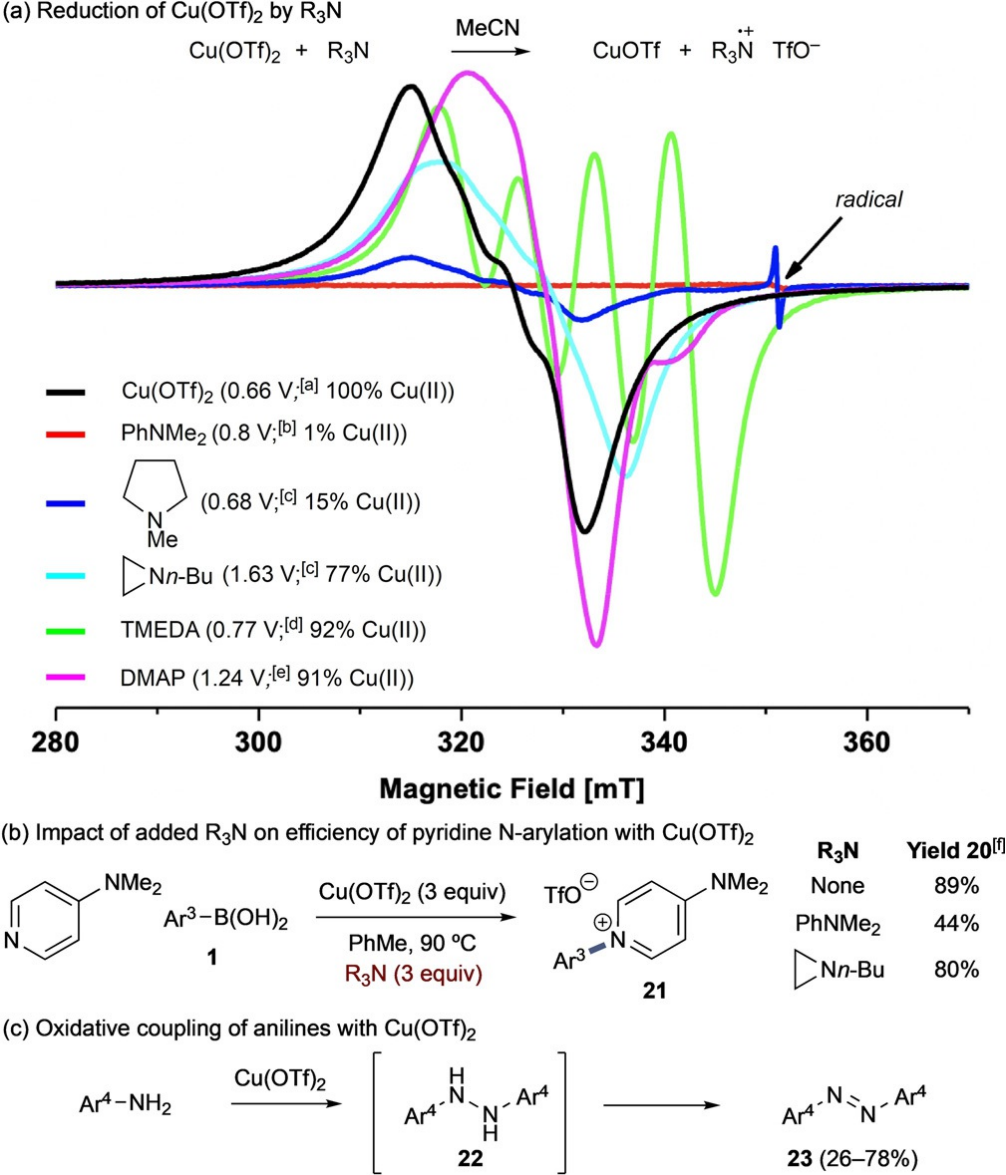
Limitations of the Cu‐mediated arylation with R_3_N. Ar^3^=4‐MeC_6_H_4_. Ar^4^=4‐FC_6_H_4_. [a] vs. Fc^+/0^.^[39]^ [b] vs. SCE.^[40]^ [c] vs. SCE.^[41]^ [d] vs. SCE.^[42]^ [e] vs. SCE.^[43]^ [f] Determined by ^1^H NMR analysis. Fc=ferrocene, SCE=saturated calomel electrode.

Despite evidence for the feasibility of reductive elimination from (aryl)Cu^III^ complexes yielding N‐aryl ammonium products,[^36^, ^37^] the equivalent N(sp^3^) cross‐coupling under the conditions reported here did not afford the desired C‐N(sp^3^) bond. We attribute this to competing amine oxidation by Cu^II^;^[38]^ this was substantiated by EPR studies, which showed quenching of Cu^II^ and, in the case of N‐methylpyrrolidine, a radical species could be observed (Scheme 6 a). Addition of tertiary amines to the optimized DMAP N‐arylation process had variable effects on the observed yield (Scheme 6 b). For example, PhNMe_2_ almost completely reduced Cu^II^ and lowered yield of **21** by approximately half; however, *n*‐butylaziridine reduced approx. 25 % of Cu^II^ yet had no impact on the yield of **21**. Little reduction of Cu^II^ by TMEDA was observed by EPR and the arylation reaction was instead impaired by formation of a series of novel but unreactive bidentate complexes (Scheme S12). As expected, DMAP did not significantly reduce Cu^II^.

Moreover, in the presence of unsubstituted anilines, an alternative oxidative coupling pathway becomes evident via formation of 1,2‐diarylhydrazines (**22**) and azobenzenes (**23**) (Scheme 6 c). This is clearly mechanistically related to previously reported Cu‐mediated N‐N coupling reactions.[^44^, ^45^] Consistent with these previous reports, our EPR data suggests that these processes proceed via single electron oxidation of the aniline by Cu^II^; however, importantly, the resulting aminium radical does not appear to be free in solution and attempts to intercept these species were universally unsuccessful (Table S6). In contrast to a previously proposed mechanism,^[44]^ our data suggests formation of the N−N bond at the metal or within the solvent cage. This would deliver the symmetrical hydrazine product, consistent with previous observations.[^44^, ^45^] As an adjunct to the main work described here, additional control experiments have shown facile oxidation of the hydrazine to the azobenzene by Cu(OTf)_2_ aligning with the experimental data observed across these separate studies (Scheme S14).[^44^, ^45^]

**Scheme 7:**
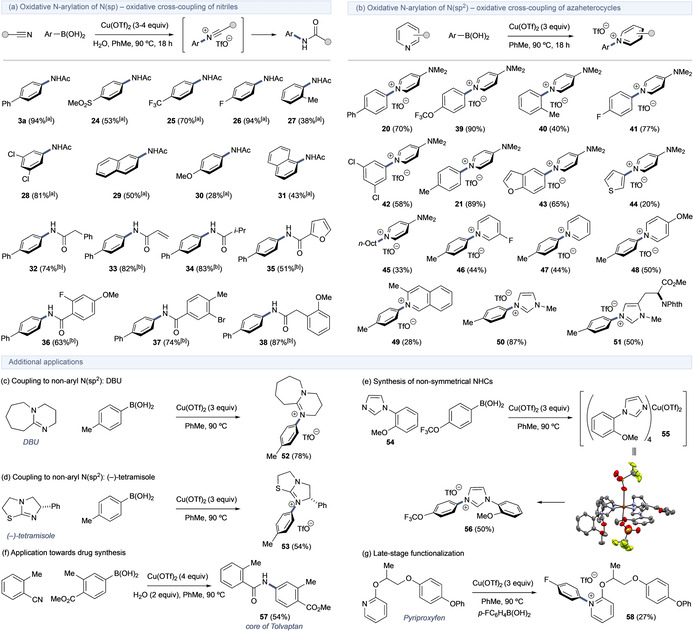
a) Cu‐mediated N‐arylation of N(sp) and N(sp^2^): representative examples. [a] Cu(OTf)_2_ (3 equiv), H_2_O (3 equiv), MeCN as solvent. [b] Cu(OTf)_2_ (4 equiv), H_2_O (2 equiv), PhMe as solvent. Ac=acetyl, Phth=phthalimide.

Following optimization (Tables S7–S11), a general process was developed for the coupling of arylboronic acids with nitriles and N‐heterocycles—a selection of products is provided in Scheme 7 (for additional substrates see Scheme S15).^[46]^ The process tolerates a variety of functional groups on both the nitrile and arylboronic acid, with standard structural and electronic variations examined in this example scope. The nitrilium process is an unusual amidation protocol (essentially an aryl Ritter reaction) providing a new approach to this ubiquitous motif; however, the heterocycle N‐arylation process allows access to products that cannot be made easily using any established method, providing novel opportunities for synthetic design. In general, the scope of the boronic acid was very good for arylboronic acids, with some lower yields observed using heteroaromatic species consistent with established limitations with these substrates.^[47]^ Alkylboronic acids were tolerated only in the N‐heterocycle process (e.g., product **45**); no desired products were observed in the equivalent nitrilium reactions. For the nitrilium process, the C‐N cross‐coupling could be achieved using the nitrile as solvent where practical (e.g., for MeCN, EtCN), otherwise PhMe was the preferred medium for both the nitrilium and N‐heterocycle processes. While generally effective, solubility issues can present with certain arylboronic acids in PhMe resulting in lower yields (e.g., **29**–**31**). With regards the N‐heterocycle process, the reaction was broadly tolerant to the nature of the heterocycle, although higher yields were obtained with more electron‐rich compounds, which may be expected based on the oxidative coupling process. The issue of lower yields with substrates bearing *ortho*‐substitution was replicated (e.g., **27** and **40**) and is again consistent with observations in Cu‐mediated oxidative coupling processes.^[9]^ As discussed above for the nitrile process, stoichiometric Cu(OTf)_2_ was also needed for the heterocycle process, which perhaps offers some explanation for the lack of observable reinsertion into the N‐aryl pyridinium products. Additional demonstrations of utility are provided in Scheme 7 c‐g. The C‐N coupling process can be applied to the N‐arylation of non‐aryl N(sp^2^) including the common organic base DBU as well as the Lewis base organocatalyst (−)‐tetramisole to afford compounds **52** and **53**, respectively (Schemes 7 c and d).

The ability to induce direct N‐arylation of N‐heterocycles allows a significantly shorter route to non‐symmetrical NHCs by N‐arylation of N‐aryl imidazoles such as **54**, which proceeds via the expected complex **55** to deliver imidazolium salt **56** (Scheme 7 e; see also **50** and **51** in Scheme 7 b for alkyl/aryl imidazolium).^[11]^ Lastly, the process can be used in synthesis, for example using the nitrilium process to access pharmaceutically relevant amides, such as the Tolvaptan intermediate **57** (Scheme 7 f) and for late‐stage functionalization, for example N‐arylation of the agrochemical Pyriproxyfen, giving product **58** (Scheme 7 g).

## Conclusion

In summary, the data provided establishes a framework for oxidative C‐N cross‐coupling of arylboronic acids with neutral N‐ligands. Importantly, mechanistic data supports a Cu^III^‐based process and is distinct from Lewis acid‐assisted N‐arylations using reactive aryl transfer electrophiles (e.g., iodoniums). This expands the scope of oxidative coupling, allowing access to new products. The broader implications are that, assuming specific metal‐centered mechanistic events can be appropriately controlled, neutral N‐ligands may be effective partners for cross‐coupling more generally within transition metal catalysis, providing new opportunities for reaction design.^[48]^

## Conflict of interest

The authors declare no conflict of interest.

## Supporting information

As a service to our authors and readers, this journal provides supporting information supplied by the authors. Such materials are peer reviewed and may be re‐organized for online delivery, but are not copy‐edited or typeset. Technical support issues arising from supporting information (other than missing files) should be addressed to the authors.

Supplementary

Supplementary
