# Supplementary material for: Cu(OTf)2‐Mediated Cross‐Coupling of Nitriles and N‐Heterocycles with Arylboronic Acids to Generate Nitrilium and Pyridinium Products
Source: Angew Chem Int Ed Engl. 2021 Feb 26;60(14):7935–40. doi: 10.1002/anie.202016811 (PMC8048606; doi:10.1002/anie.202016811)
Supplement: Supplementary file 2 — Supplementary [file ANIE-60-7935-s002.pdf]

Angewandte Chemie
Eine Zeitschrift der Gesellschaft Deutscher Chemiker

Supporting Information

$Cu(OTf)_2$-Mediated Cross-Coupling of Nitriles and N-Heterocycles with Arylboronic Acids to Generate Nitrilium and Pyridinium Products**

*Nicola L. Bell[+], Chao Xu[+], James W. B. Fyfe, Julien C. Vantourout, Jeremy Brals, Sonia Chabbra, Bela E. Bode, David B. Cordes, Alexandra M. Z. Slawin, Thomas M. McGuire, and Allan J. B. Watson**

anie_202016811_sm_miscellaneous_information.pdf
anie_202016811_sm_miscellaneous_information.cif

Table of Contents

1. General Information *3*

2. General Experimental Procedures *5*

3. Preparation of Reaction-relevant Species *7*

4. Control Experiments *9*

5. Optimization Studies *25*

6. Experimental Procedures and Characterization Data *32*

7. X-ray Crystallography *61*

8. References *64*

1. General Information

Purification of Solvents & Reagents

Dry solvent (PhMe) for reactions was obtained from a PureSolv SPS-400-5 solvent purification system. MeCN was dried by heating to reflux over CaH_2 and distilling under reduced pressure before being purged with, and stored under N_2 in a J-Youngs tapped, oven-dried flask over previously activated 3 Å molecular sieves. DCM, MeCN, Et_2O, EtOAc, and petroleum ether 40-60 °C for purification purposes were used as obtained from suppliers without further purification.

Copper(II) triflate was purchased from either TCI Europe or Sigma-Aldrich and stored in an argon-filled glovebox at all times. The $Cu(OTf)_2$ must be a white powder. Boronic acids were purified by crystallization from acetone/H_2O (1:2). Pyridine, 4-methoxypyridine, and DBU were heated over CaH_2, distilled, purged with and stored under N_2 over molecular sieves prior to use. Tetramisole was obtained by basic extraction from Tetramisole·HCl, purchased from Sigma-Aldrich.

All other reagents and solvents were obtained from commercial suppliers and were used without further purification unless otherwise stated. Purification was carried out according to standard laboratory methods.

Experimental Details

Reactions were carried out using conventional glassware (preparation of intermediates) or in capped 5 mL microwave vials. The glassware was oven-dried (150 °C) and transferred to an argon filled glovebox whilst hot. Reaction mixtures were prepared in the glovebox with H_2O added through a septum outside the box, where required. Room temperature was generally *ca.* 18 °C. Reactions were carried out at elevated temperatures in a sand bath atop a temperature-regulated hotplate/stirrer. All reactions were carried out with 8 mm magnetic stirring bars.

Purification of Products

For aryl amide products (Scheme 7a), thin layer chromatography was carried out using Merck silica plates coated with fluorescent indicator UV254. These were analyzed under 254 nm UV light or developed using potassium permanganate solution. Normal phase flash chromatography was

carried out using ZEOprep 60 HYD 40-63 μm silica gel. For pyridinium products (Scheme 7b), thin layer chromatography was carried out on Sigma-Aldrich Basic alumina plates with fluorescent indicator UV254. These were analyzed under 254 nm UV light. Normal phase flash chromatography was carried out using Brockmann 1 basic alumina gel, standard grade, ~150 mesh, 58 Å.

Analysis of Products

Fourier Transformed Infra-Red (FTIR) spectra were obtained on a Shimadzu IRAffinity-1 machine. ^{1}H and ^{13}C NMR spectra were obtained on either a Bruker AV 400 at 400 MHz and 101 MHz, respectively, or Bruker DRX 500 at 500 MHz and 126 MHz, respectively. ^{19}F NMR spectra were obtained on a Bruker AV 400 spectrometer at 376 MHz. ^{11}B NMR spectra were obtained on a Bruker AV 400 spectrometer at 128 MHz. Chemical shifts are reported in ppm and coupling constants are reported in Hz with $CDCl_3$ referenced at 7.26 (^{1}H) and 77.16 ppm (^{13}C), DMSO-d_6 referenced at 2.50 (^{1}H) and 39.5 (^{13}C) and CD_3OD referenced at 3.31 (^{1}H) and 49.0 (^{13}C). ^{11}B NMR spectra are referenced to $BF_3 \cdot Et_2O$. High-resolution mass spectra were obtained through analysis at the EPSRC UK National Mass Spectrometry Facility at Swansea University and at the University of St Andrews. NMR assays were conducted with dibenzylether as an internal standard (0.25 M solution in DCM). Reverse phase HPLC data was obtained on an Agilent 1200 series HPLC using a Machery-Nagel Nucleodur C18 column. Samples for HPLC analysis were prepared through the addition of 2 mL of caffeine standard to the completed reaction mixture before analysis against established conversion factors. Elemental analyses were carried out by Dr Stephen Boyer at London Metropolitan University.

2. General Experimental Procedures

General Procedure A: Synthesis of amide products via nitrile *N*-arylation with MeCN

An oven dried 5 mL microwave vial containing a stirrer bar was charged with arylboronic acid (1.0 equiv.) and $Cu(OTf)_2$ (3.0 equiv.). The microwave vial was capped and purged with N_2 before adding MeCN (0.25 M) and H_2O (3.0 equiv.) The stirred reaction mixture was then heated to 90 °C for 3 h. The reaction mixture was cooled to room temperature and saturated aqueous ammonia solution (3.0 mL/mmol Cu) and brine (10 mL/mmol Cu) were added to the crude reaction mixture. The aqueous phase was then extracted with EtOAc (2 × 10 mL) and the combined organic phase was dried over Na_2SO_4, passed through a hydrophobic frit, and concentrated under reduced pressure. The crude mixture was purified with flash chromatography (silica gel, EtOAc/petroleum ether) to afford the desired product.

General Procedure B: Synthesis of amide products via nitrile *N*-arylation with other nitriles

An oven dried 5 mL microwave vial containing a stirrer bar was charged with the arylboronic acid (1.0 equiv.) and $Cu(OTf)_2$ (4.0 equiv.) before adding solvent (toluene unless otherwise stated, 0.25 M) followed by nitrile (10.0 equiv). The microwave vial was capped and H_2O (2 equiv.) was added before the stirred reaction mixture was then heated to 90 °C for *ca.* 16 h. The reaction mixture was cooled to room temperature and saturated aqueous ammonia solution (3.0 mL/mmol Cu) and brine (10 mL/mmol Cu) were added to the crude reaction mixture. The aqueous phase was then extracted with DCM and the combined organic phase was dried over Na_2SO_4, passed through a glass fiber filter plug, and concentrated under reduced pressure. The crude mixture was purified with flash chromatography (silica gel, EtOAc/petroleum ether) to afford the desired product.

General Procedure C: Synthesis of pyridinium products via *N*-arylation

In an argon filled glovebox, an oven dried 5 mL microwave vial was charged with arylboronic acid (1.0 equiv.), and $Cu(OTf)_2$ (3.0 equiv.) before addition of solvent (toluene unless otherwise stated, 0.25 M). The reagents were stirred for at least 1 h in an argon filled glovebox before addition of the N-heterocycle (5.0 equiv.). The microwave vial was capped, removed from the glovebox, and the stirred reaction mixture then heated to 90 °C for 16 h. The reaction mixture was cooled to room temperature and DCM was added (along with 1 equiv. dibenzyl ether as an internal standard, where appropriate). The suspension was filtered through fluted filter paper and

dried under reduced pressure. The crude mixture was purified with flash chromatography (basic alumina, DCM:MeCN, 1:1) to afford the desired product. Note: products were prone to degradation on both silica and alumina; however, rapid alumina purification allowed isolation.

3. Preparation of Reaction-relevant Species

Scheme S1: Synthesis of Cu complex 10a

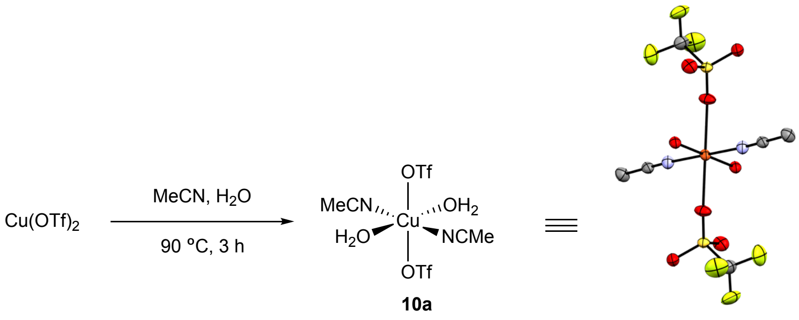

$Cu(OTf)_2$ (1.0 g, 2.8 mmol, 1 equiv.) was added to an oven dried round bottom flask. The flask was sealed and purged with N_2 before adding freshly distilled MeCN (4 mL) and H_2O (100 µL, 5.6 mmol, 2 equiv.). The mixture was heated at 90 °C for 3 h, before cooling to room temperature. The resulting homogenous blue solution was transferred to a 15 mL vial and capped with a pierced lid and allowed to slowly evaporate to afford single crystals of complex **10a** as blue solid which was collected by filtration (500 mg, 52%). The structure of **10a** was confirmed by X-ray analysis and the unit cell was in agreement with that of the known structure (CSD 601802).[1]

Scheme S2: Synthesis of Cu complex 10b

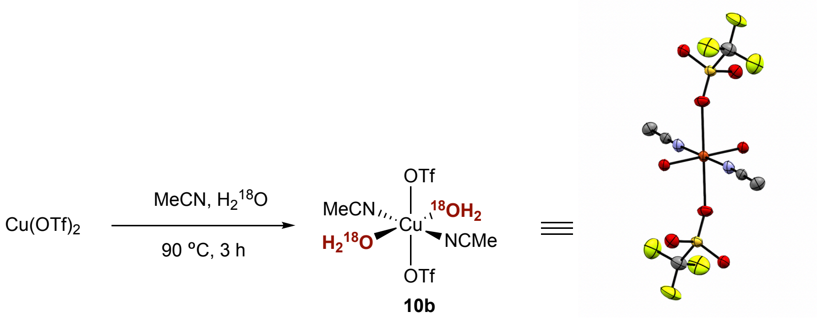

In a glovebox, $Cu(OTf)_2$ (1.0 g, 2.8 mmol, 1 equiv.) was added to a flame dried round bottom flask. The flask was sealed and removed from the glovebox before adding freshly distilled MeCN (4 mL) and $H_2{}^{18}O$ (100 µL, 4.5 mmol, 1.6 equiv.). The mixture was heated at 90 °C for 3 h, before cooling to room temperature. The flask was then moved into a glovebox and the resulting homogenous blue solution was transferred to a 15 mL vial and capped with a pierced lid and allowed to slowly evaporate to afford single crystals of complex **10b** as blue solid which was

collected by filtration (1.1 g, >99%). The structure of **10b** was confirmed by X-ray analysis and the unit cell was in agreement with that of the known structure (CSD 601802).[2]

Scheme S3: Synthesis of Cu complex 19

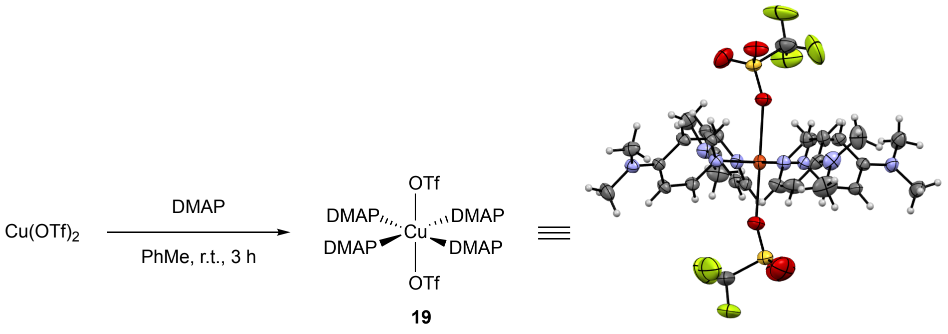

$Cu(OTf)_2$ (200 mg, 0.55 mmol, 1 equiv.) was suspended in PhMe (ca. 10 mL) before addition of 4-dimethylaminopyridine (270 mg, 2.21 mmol, 4 equiv.). The purple suspension was stirred at room temperature for 3 h before filtration to give a purple solid (450 mg, 96%). This solid was dissolved in a small quantity of $CHCl_3$ and single crystals were obtained by vapor diffusion with hexanes to afford complex **19** as a purple solid. The structure of **19** was confirmed by X-ray analysis (CSD 1971679).

Elemental Analysis: $C_{30}H_{40}N_8O_6F_6S_2Cu$ requires C, 42.36; H, 4.74; N, 13.18%; found C, 42.58; H, 4.47; N, 12.92%

*NB: Following confirmation of structure by X-ray analysis, single crystal complexes **10a**, **10b**, and **19** were ground with mortar and pestle before use in subsequent reactions.*

4. Control Experiments

Scheme S4: Chan-Lam reaction pathway control reaction (Scheme 3a)

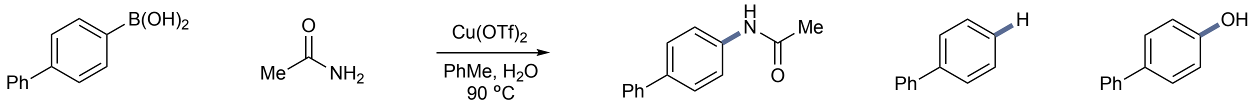

An oven dried 5 mL microwave vial was charged with 4-biphenylboronic acid (50 mg, 0.25 mmol, 1.0 equiv.), acetamide (74 mg, 1.25 mmol, 5.0 equiv.), and $Cu(OTf)_2$ (360 mg, 1 mmol, 4.0 equiv.). The vial was then capped before adding H_2O (9 µL, 0.5 mmol, 2 equiv.) and PhMe (1 mL, 0.25 M). The mixture was stirred at 90 °C for 16 h. Conversion was determined by HPLC with the use of caffeine as an internal standard.

No Chan-Lam amination product was observed.

Table S1: Hydrolysis of nitrile under reaction conditions

An oven dried 5 mL microwave vial was charged with 2-phenylacetonitrile (29 mg, 0.25 mmol, 1 equiv.), Cu catalyst (2 equiv.), additive (1 equiv.), H_2O (X equiv.), and PhMe (0.25 M). The mixture was stirred at 90 °C for 16 h. The reaction mixture was diluted with EtOAc (10 mL) and washed with sat. aq. $NaHCO_3$ (10 mL) and brine (10 mL). The aqueous phase was then extracted with EtOAc (10 mL) and the combined organic phase was dried over Na_2SO_4 and concentrated under reduced pressure. The crude material was then analyzed by ^1H NMR.

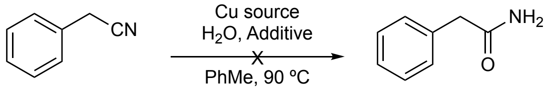

Entry	Cu (2 equiv.)	H_2O (equiv.)	Additives (1 equiv.)	Yield
1	$Cu(OTf)_2$	2	$B(OH)_3$	0%
2	$Cu(OTf)_2$	10	-	0%
3	$Cu(OTf)_2$	2	TfOH	0%
4	$Cu(OTf)_2$	2	AcOH	0%
5	Cu(OTf)·PhMe	2	-	0%
6	Cu(OTf)·PhMe	2	TfOH	0%

7	CuTC	5	-	0%
8	CuTC	5	$B(OH)_3$	0%
9	CuTC	5	TfOH	0%
10	CuI	5	TfOH	0%

No hydrolysis was observed under any reaction conditions.

Scheme S5: Competition experiment between nitrile and acetamide (Scheme 3b)

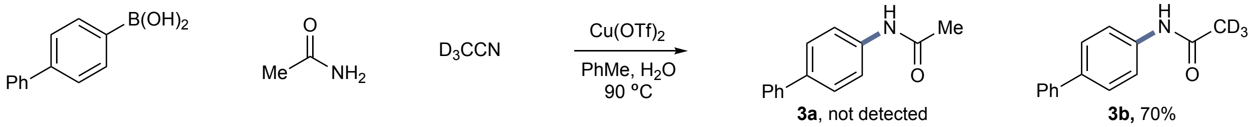

An oven dried 5 mL microwave vial was charged with 4-biphenylboronic acid (50 mg, 0.25 mmol, 1.0 equiv.), acetamide (74 mg, 1.25 mmol, 5.0 equiv.), and $Cu(OTf)_2$ (360 mg, 1 mmol, 4.0 equiv.). The vial was then capped before adding H_2O (9 µL, 0.5 mmol, 2 equiv.), d_3-MeCN (65 µL, 1.25 mmol, 5.0 equiv.), and PhMe (1 mL, 0.25 M). The mixture was stirred at 90 °C for 16 h. Saturated aqueous ammonia solution (10 mL) and brine (10 mL) were added to the crude reaction mixture. The aqueous phase was then extracted with EtOAc (2 × 10 mL) and the combined organic phase was dried over Na_2SO_4, and concentrated under reduced pressure. The crude mixture was purified with flash chromatography (silica gel, 10-30% EtOAc/petroleum ether) to afford the desired product as a beige solid (37 mg, 70%). Deuterium incorporation was confirmed by ^1H and ^2H NMR.
No Chan-Lam product was observed; exclusive reaction of the nitrile.

Scheme S6: Synthesis of amide 3a from complex 10a (Scheme 3c)

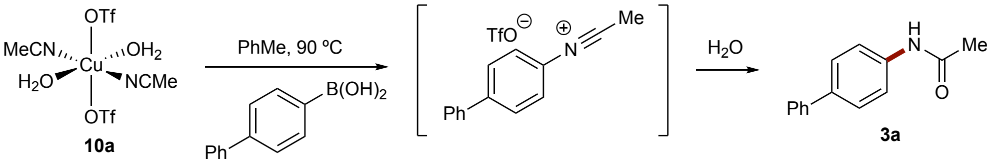

In an argon filled glovebox, an oven dried 5 mL microwave vial was charged with 4-biphenylboronic acid (25 mg, 0.125 mmol, 1.0 equiv.) and complex **10a** (121 mg, 0.25 mmol, 2.0 equiv.) in PhMe (0.5 mL, 0.25 M). The microwave vial was capped, removed from the glovebox,

and the reaction mixture then heated to 90 °C for 18 h. The reaction mixture was cooled to room temperature and saturated aqueous ammonia solution (10 mL) and brine (10 mL) were added to the crude reaction mixture. The aqueous phase was then extracted with EtOAc (2 × 10 mL) and the combined organic phase was dried over Na_2SO_4, filtered, and concentrated under reduced pressure. The crude mixture was purified with flash chromatography (silica gel, EtOAc/petroleum ether, 10-30%) to afford the desired product as a beige solid (8 mg, 31%).

Attempts at intra- and intermolecular traps of nitrilium intermediate

Intra- and intermolecular trap reactions were run according to either General Procedure A or B. Crude reaction mixtures were analyzed by ^{1}H NMR and then purified by column chromatography (silica gel).

Table S2: Intramolecular Traps

General Procedure	Boronic Acid	Nitrile	Desired Product	Successful (Y/N)
A	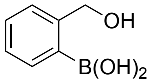	MeCN	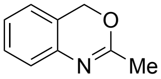	N
A	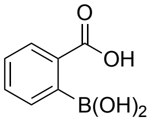	MeCN	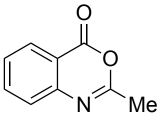	N
A	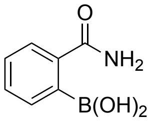	MeCN	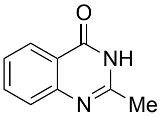	N
A	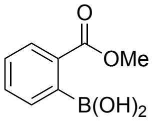	MeCN	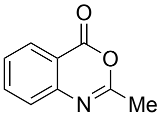	N
B	$PhB(OH)_2$	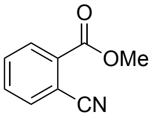	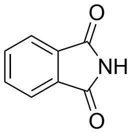	N
B	$PhB(OH)_2$	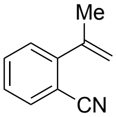	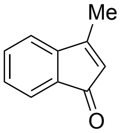	N
B	$PhB(OH)_2$	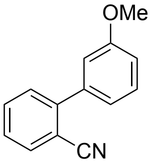	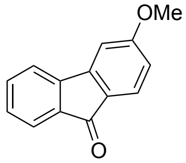	N
B	$TolylB(OH)_2$	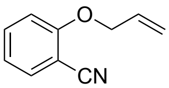	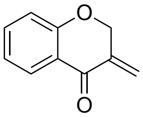	N
B	$TolylB(OH)_2$	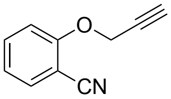	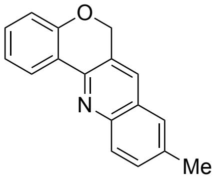	N

B*	$(TolylBO)_3$	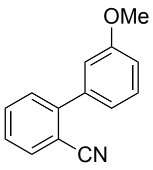	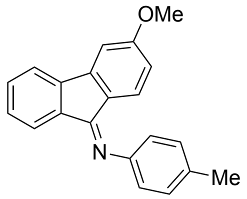	N
B*	$(TolylBO)_3$	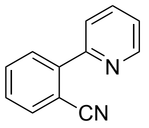	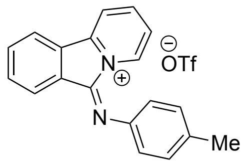	N

*Reactions using 4-tolylboroxine were run with either NaOAc or NaOMe (3 equiv.) as an activator.

In all cases the desired product of intramolecular trapping of the nitrilium intermediate were not observed or isolated.

Table S3: Intermolecular Traps

General Procedure	Boronic Acid	Nitrile	Nucleophile (5 equiv.)	Desired Product	Successful (Y/N)
B	$PhB(OH)_2$	MeCN	MeOH	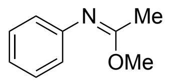	N
B	$PhB(OH)_2$	MeCN	KF	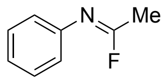	N
B	$PhB(OH)_2$	MeCN	Xylene	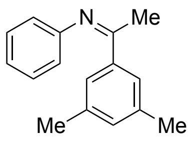	N
B	$TolylB(OH)_2$	MeCN	DMAP	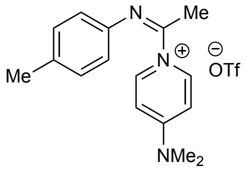	N
B	$TolylB(OH)_2$	MeCN	DMAP + 3 equiv. H_2O	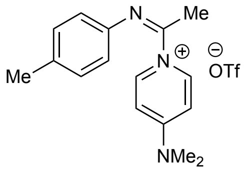	N
B	$(TolylBO)_3$	MeCN	MeOH	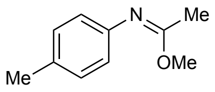	N
B	$(TolylBO)_3$	MeCN	MeOH + 3 equiv. H_2O	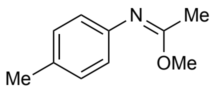	N
B	$(TolylBO)_3$	MeCN	DMAP	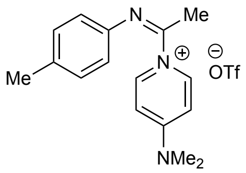	N
B	$(TolylBO)_3$	MeCN	DMAP + 3 equiv. H_2O	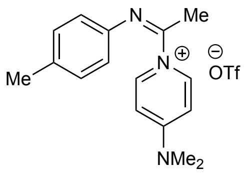	N

In all cases the desired product of intermolecular trapping of the nitrilium intermediate were not observed or isolated.

Scheme S7: Rapid hydrolysis of nitrilium (Scheme 3d)

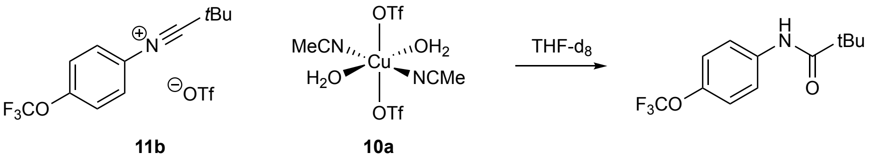

In an argon filled glovebox, nitrillium **11b** (10 mg, 0.025 mmol, 1 equiv.) (prepared from the corresponding pivalamide according to procedure by Lammertsma et al, *Angew. Chem. Int. Ed.* **2014**, *53*, 9068) and complex **10a** (14.3 mg, 0.03 mmol, 1.2 equiv.) were added to an NMR tube and dissolved in dry THF-d_8. The tube was then capped and removed from the glovebox and analyzed by ^{1}H NMR.

Complete conversion to the corresponding amide product was observed.

Scheme S8: Synthesis of amide 3c from complex 10a and $H_2{}^{18}O$ (Scheme 3e)

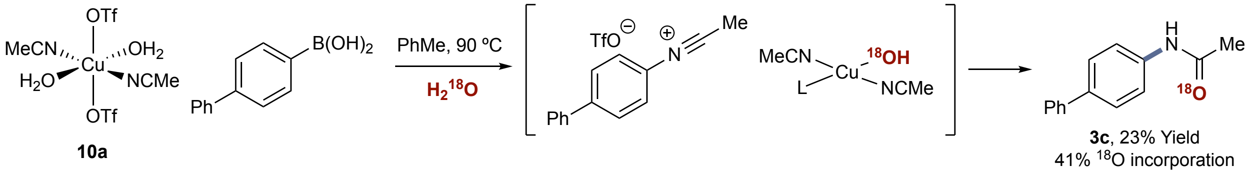

Reaction carried out according to General Procedure B using 4-biphenylboronic acid (25 mg, 0.125 mmol, 1.0 equiv.), complex **10a** (121 mg, 0.25 mmol, 2.0 equiv.) and $H_2{}^{18}O$ (6.5 µL, 0.375 mmol, 3.0 equiv.) in PhMe (0.5 mL, 0.25 M). The microwave vial was capped under air and the reaction mixture then heated to 90 °C for 18 h. The reaction mixture was subjected to the purification outlined in the General Procedure (silica gel, 10-50% EtOAc/petroleum ether) to afford the desired product as a white solid (6 mg, 23%).

Scheme S9: Synthesis of amide 3c from ^{18}O labelled complex 10b (Scheme 3f)

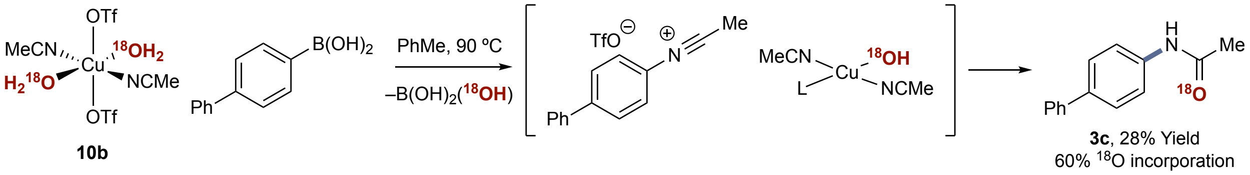

In an argon filled glovebox, the reaction carried out according to General Procedure B using 4-biphenylboronic acid (50 mg, 0.25 mmol, 1.0 equiv.) and complex **10b** (242 mg, 0.5 mmol, 2.0 equiv.) in PhMe (1 mL, 0.25 M). The microwave vial was capped, removed from the glovebox and the reaction mixture then heated to 90 °C for 18 h. The reaction mixture was subjected to the purification outlined in the General Procedure (silica gel, 10-50% EtOAc/petroleum ether) to afford the desired product as a white solid (15 mg, 28%).

Figure S1: HRMS study of reaction mixture at r.t.

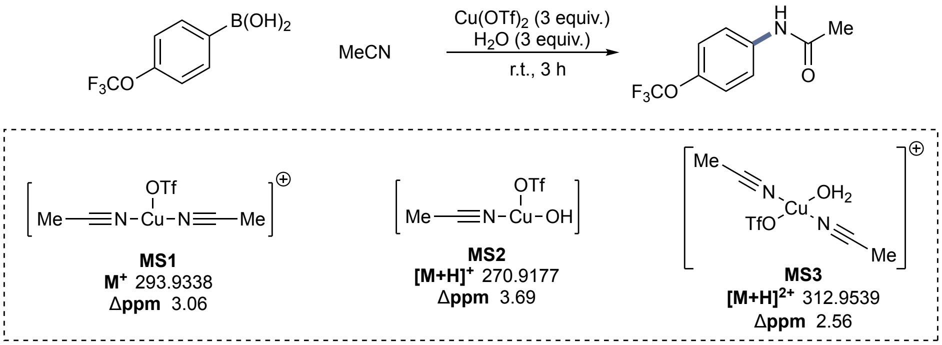

Reaction carried out according to General Procedure A using (4-(trifluoromethoxy)phenyl)boronic acid (51.5 mg, 0.25 mmol, 1 equiv.), $Cu(OTf)_2$ (271.2 mg, 0.75 mmol, 3 equiv.), and H_2O (13.5 µL, 0.75 mmol, 3 equiv.) in MeCN (0.25 M). The resulting mixture was stirred at room temperature for 3 h before an aliquot was taken and subjected to HRMS analysis (ESI).

Mass ions consistent with the above structures were detected.

Figure S2: HRMS study of reaction mixture at 90 °C

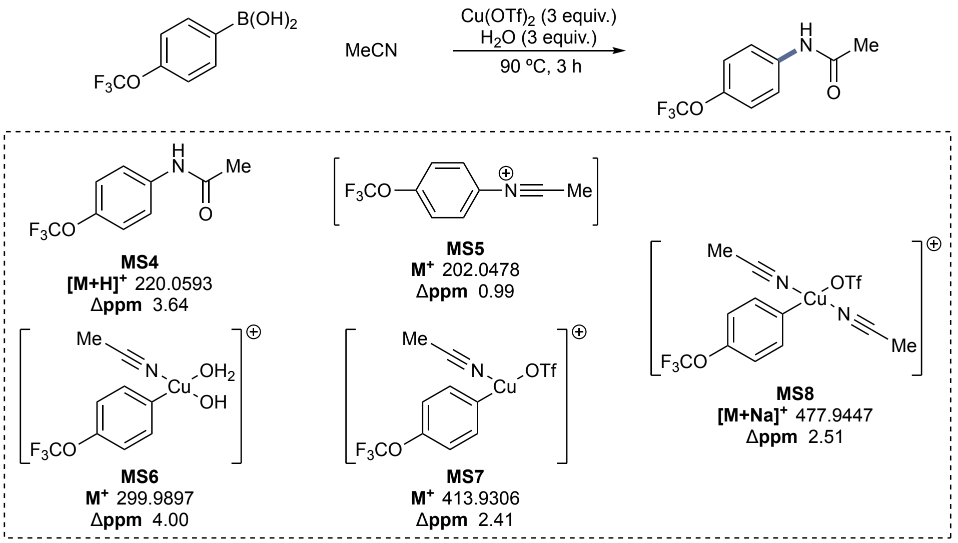

Reaction carried out according to General Procedure A using (4-(trifluoromethoxy)phenyl)boronic acid (51.5 mg, 0.25 mmol, 1 equiv.), $Cu(OTf)_2$ (271.2 mg, 0.75 mmol, 3 equiv.), and H_2O (13.5 µL, 0.75 mmol, 3 equiv.) in MeCN (0.25 M). The resulting mixture was stirred at 90 °C for 3 h before an aliquot was taken and subjected to HRMS analysis (ESI).

Mass ions consistent with above structures were detected.

Table S4: Theoretical and observed mass ions from Figure S1 and S2

Compound	Formula	Theoretical *m/z*	Observed *m/z*
**MS1**	$C_5H_6CuF_3N_2O_3S$	293.9347	293.9338
**MS2**	$C_3H_5CuF_3NO_4S$	270.9187	270.9177
**MS3**	$C_5H_9CuF_3N_2O_4S$	312.9531	312.9539
**MS4**	$C_9H_9F_3NO_2$	220.0585	220.0593
**MS5**	$C_9H_7F_3NO$	202.0480	202.0478
**MS6**	$C_9H_{10}CuF_3NO_3$	299.9909	299.9897
**MS7**	$C_{10}H_7CuF_6NO_4S$	413.9296	413.9306
**MS8**	$C_{12}H_{10}CuF_6N_2O_4SNa$	477.9459	477.9447

Scheme S10: Synthesis of pyridinium 20 from complex 19 (Scheme 5)

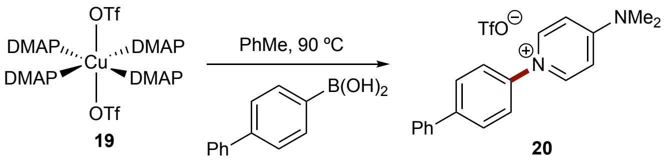

In an argon filled glovebox, an oven dried 5 mL microwave vial was charged with 4-biphenylboronic acid (50 mg, 0.25 mmol, 1.0 equiv.), and complex **19** (425 mg, 0.5 mmol, 2.0 equiv.) before addition of toluene (1.0 mL, 0.25 M). The microwave vial was capped, removed

from the glovebox, and the reaction mixture was then heated to 90 °C for 18 h. The reaction mixture was cooled to room temperature and DCM was added. The suspension was filtered through fluted filter paper and dried under reduced pressure. The crude mixture was purified with flash chromatography (basic alumina, DCM:MeCN, 1:1) to afford the desired product as a white solid (27 mg, 27%).

Scheme S11: EPR analysis of $Cu(OTf)_2$ with tertiary amines

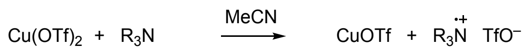

Figure S3: EPR spectra of $Cu(OTf)_2$ with tertiary amines (Scheme 6a)

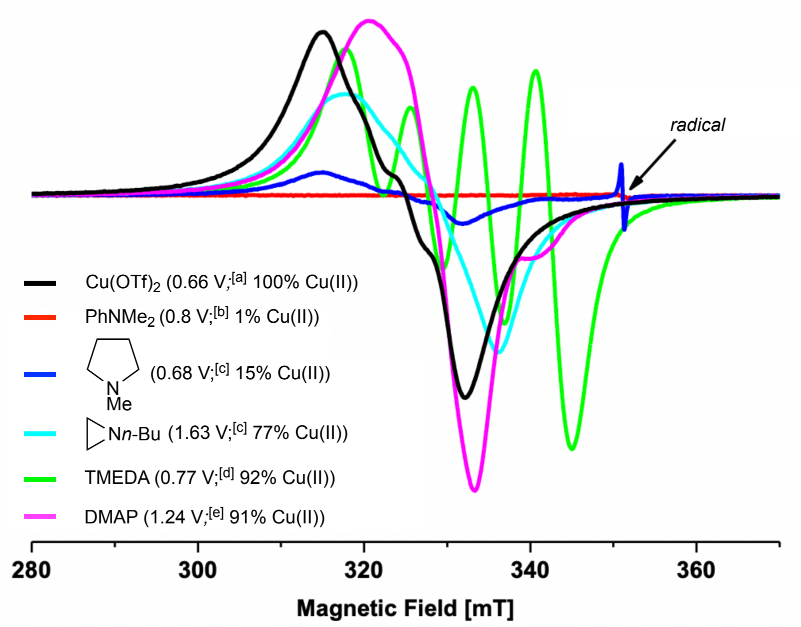

X-band cw EPR spectra of $Cu(OTf)_2$ with tertiary amines at 295 K. The relative double integrals with respect to the $Cu(OTf)_2$ are expressed as a percentage in the legend for all the samples.

X-band cw EPR spectra were obtained at 295 K for $Cu(OTf)_2$ with and without tertiary amines under identical conditions (Fig 1). Addition of DMAP to the $Cu(OTf)_2$ led to ~91% EPR signal recovery showing formation of $Cu(II)(OTf)_2(DMAP)_4$ which was found to be active for N-arylation as shown in the main text. In contrast to DMAP, other tertiary amine complexes displayed reduction in the relative amount of Cu(II) with decreasing oxidation potential of the amine, demonstrating that substrate oxidation is a key competing pathway preventing ionizing cross coupling for amines. In the case of N-Methyl pyrrolidine, a radical species was detected showing quenching of Cu(II). In contrast, TMEDA disfavors Cu(II) reduction due to the chelate effect and ~92% signal was recovered due to the formation of a series of unreactive $Cu(OTf)_2$•TMEDA complexes.

Scheme S12: $Cu(OTf)_2$•TMEDA complexes

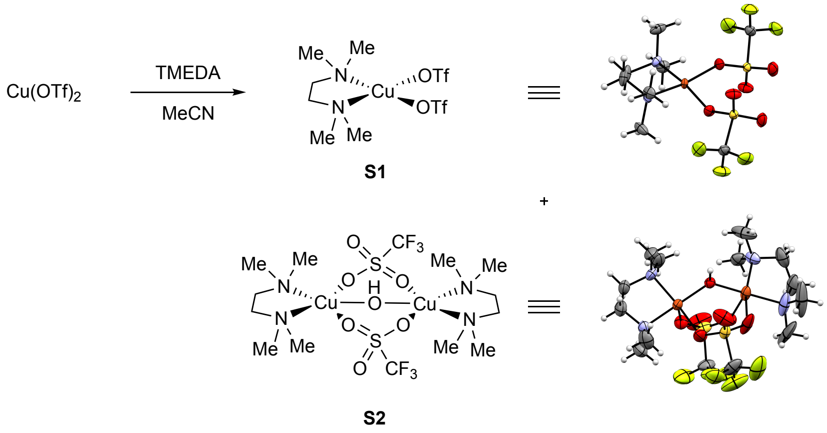

Complexes **S1** and **S2** were isolated from a solution of $Cu(OTf)_2$ and TMEDA in MeCN following vapour diffusion with hexanes. The structures of **S1** and **S2** were confirmed by X-ray analysis (CSD 2042594 and 2042593, respectively).

Scheme S13: Inhibition of cross coupling with tertiary amines (Scheme 6b)

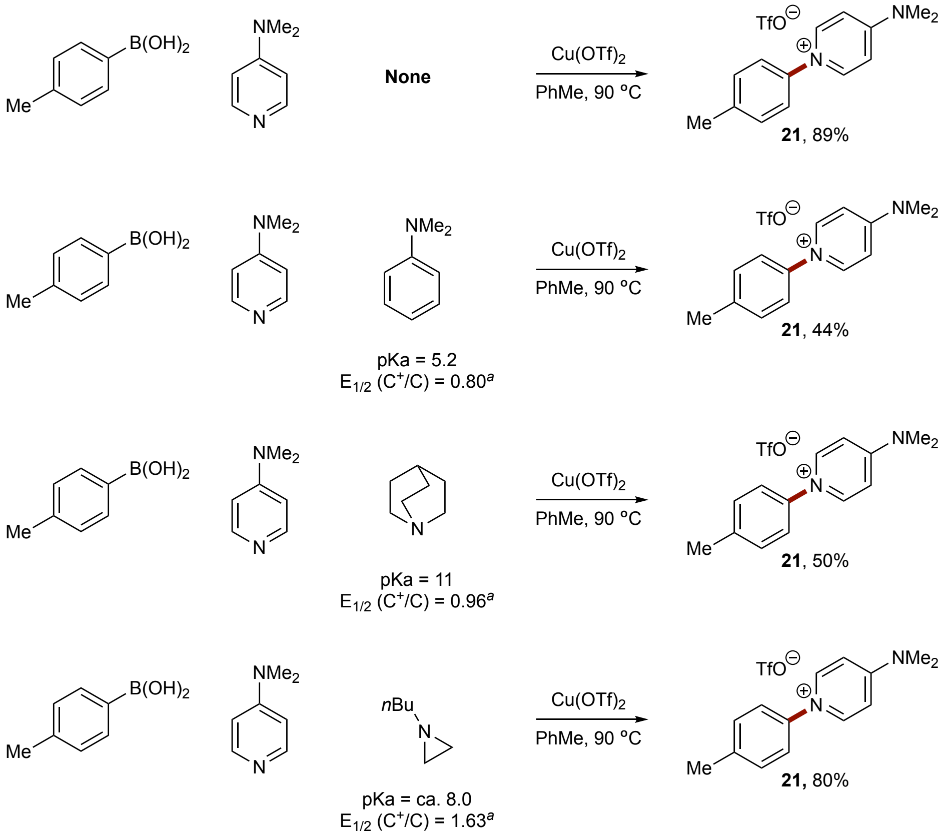

[a]$E_{1/2}$ *vs.* SCE.

Reactions carried out according to General Procedure C using 4-tolylboronic acid (34 mg, 0.25 mmol, 1.0 equiv.), $Cu(OTf)_2$ (270 mg, 0.75 mmol, 3.0 equiv.), 4-dimethylaminopyridine (153 mg, 1.25 mmol, 5.0 equiv.), and tertiary amine (3 equiv.) in PhMe (0.25 M) and stirred at 90 °C for 16 h before filtration. Conversion was determined by ^{1}H NMR with the use of dibenzylether as an internal standard.

Table S5: Oxidative coupling of anilines with $Cu(OTf)_2$ (Scheme 6c)

An oven dried 5 mL microwave vial was charged with $Cu(OTf)_2$ (90.4 mg, 0.25 mmol, 1.0 equiv.) and **Additive**. The microwave vial was capped under air before the addition of dry DCM (1 mL, 0.25 M), 4-fluoroaniline (24 µL, 0.25 mmol, 1.0 equiv.), and **base** (0.5 mmol, 2.0 equiv.). The reaction was stirred for 24 hours at room temperature under air (a fresh balloon of air was fitted on the top of each vial). After 24 hours, trifluorotoluene (30.7 µL, 0.25 mmol, 1 equiv.) was added to the reaction mixture. The reaction mixture was first quenched with 0.5 mL of a

solution of 7N ammonia in methanol and 0.5 mL of $CDCl_3$, and filtered through a celite pad. The vial was washed with 2 x 0.5 mL of $CDCl_3$ and then flushed through the pad of celite. The crude mixture was then analyzed by ^{19}F NMR (relaxation time: 10 seconds, 32 scans) and conversion against the known standard calculated.

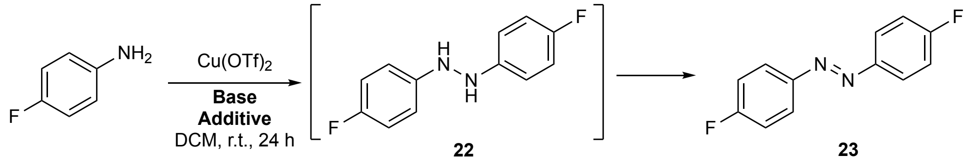

Entry	Additive (equivalents)	Base	Conversion to 23 (%)
1	None	Et_3N	26
2	None	Pyridine	2
3	None	Piperidine	32
4	None	2,6-lutidine	22
5	None	DIPEA	27
6	None	*tert*-Butylamine	42
7	None	None	0
8	Bu_4NBr (20 mol%)	Et_3N	34
9	Bu_4NBr (1 equiv.)	Et_3N	66
10	Bu_4NBr (2 equiv.)	Et_3N	66
11	LiBr (20 mol%)	Et_3N	44
12	LiBr (1 equiv.)	Et_3N	66
13	LiBr (2 equiv.)	Et_3N	78

Table S6: Radical trap experiments

An oven dried 5 mL microwave vial was charged with $Cu(OTf)_2$ (90.4 mg, 0.25 mmol, 1.0 equiv.). The microwave vial was capped under air before the addition of dry DCM (1 mL, 0.25 M), 4-fluoroaniline (24 µL, 0.25 mmol, 1.0 equiv.), styrene and Et_3N (70 µL, 0.5 mmol, 2 equiv.). The reaction was stirred for 24 hours at room temperature under air (a fresh balloon of air was fitted on the top of each vial). After 24 hours, trifluorotoluene (30.7 µL, 0.25 mmol, 1 equiv.) was added to the reaction mixture. The reaction mixture was first quenched with 0.5 mL of a solution of 7 N ammonia in methanol and 0.5 mL of $CDCl_3$, and filtered through a celite pad. The vial was washed with 2 x 0.5 mL of $CDCl_3$ and then flushed through the pad of celite. The crude mixture was then analyzed by ^{19}F NMR (relaxation time: 10 seconds, 32 scans).

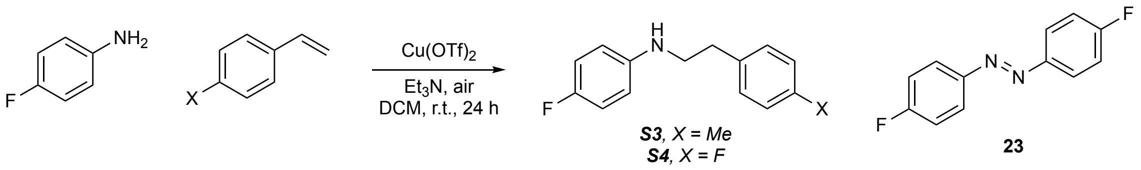

Entry	X	Equiv. of styrene	23 (%)	S3 (%)	S4 (%)
1	Me	1	30	0	-
2	Me	2	34	0	-
3	F	2	27	-	0
4	F	2	40	-	0

Scheme S14: Oxidation of hydrazine with $Cu(OTf)_2$

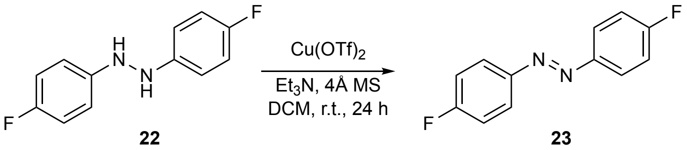

An oven dried 5 mL microwave vial was charged with $Cu(OTf)_2$ (57 mg, 0.16 mmol, 1 equiv.) along with 50 mg of oven-dried powdered 4Å molecular sieves. The microwave vial was capped under air before the addition of dry DCM (1 mL, 0.16 M), bis(4-fluorophenyl)hydrazine (35 mg, 0.16 mmol, 1 equiv.), and Et_3N (44 µL, 0.31 mmol, 2 equiv.). The reaction was stirred for 24 hours at room temperature under air (a fresh balloon of air was fitted on the top of each vial). After 24 hours, trifluorotoluene (19 µL, 0.16 mmol, 1 equiv.) was added to the reaction mixture.

The reaction mixture was first quenched with 0.5 mL of a solution of 7N ammonia in methanol and 0.5 mL of $CDCl_3$, and filtered through a celite pad. The vial was washed with 2 x 0.5 mL of $CDCl_3$ and then flushed through the pad of celite. The crude mixture was then analyzed by ^{19}F NMR (relaxation time: 10 seconds, 32 scans) and full conversion was observed (98% of diazobenzene) with no trace of SM remaining.

5. Optimization Studies

Table S7: Screening of the optimal conditions for nitrile *N*-arylation with acetonitrile

Reactions carried out according to General Procedure A using 4-biphenylboronic acid (50 mg, 0.25 mmol, 1.0 equiv.), $Cu(OTf)_2$ (a equiv.), and H_2O (b equiv.) in MeCN (0.25 M) and stirred at X °C for 3 h before aq. workup. Conversion was determined by HPLC with the use of caffeine as an internal standard.

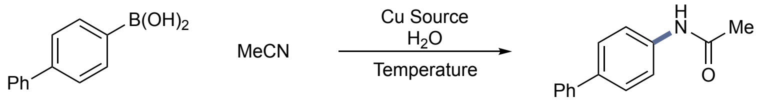

Entry	$Cu(OR)_2$	H_2O	T(°C)	Yield
**1**	$Cu(OTf)_2$ (2 equiv.)	2 equiv.	90 °C	66%
**2**	$Cu(OAc)_2$ (2 equiv.)	2 equiv.	90 °C	0%
**3**	$Cu(OAc)_2$ (2 equiv.)[a]	2 equiv.	90 °C	0%
**4**	$Cu(OTf)_2$ (2 equiv.)	2 equiv.	70 °C	0%
**5**	$Cu(OTf)_2$ (2 equiv.)	2 equiv.	80 °C	43%
**6**	$Cu(OTf)_2$ (0.2 equiv.)[b]	2 equiv.	90 °C	2%
**7**	$Cu(OTf)_2$ (1 equiv.)	2 equiv.	90 °C	31%
**8**	$Cu(OTf)_2$ (3 equiv.)	2 equiv.	90 °C	80%
**9**	$Cu(OTf)_2$ (4 equiv.)	2 equiv.	90 °C	60%
**10**	$Cu(OTf)_2$ (3 equiv.)	1 equiv.	90 °C	75%
**12**	**$Cu(OTf)_2$ (3 equiv.)**	**3 equiv.**	**90 °C**	**91%**
**13**	$Cu(OTf)_2$ (3 equiv.)	4 equiv.	90 °C	80%

[a]Using 2 equiv. TfOH as additive; [b]Under atmosphere of oxygen.

Table S8: Screening of the optimal conditions for nitrile *N*-arylation with other nitriles

Reactions carried out according to General Procedure B using 4-biphenylboronic acid (50 mg, 0.25 mmol, 1.0 equiv.), $Cu(OTf)_2$ (a equiv.), MeCN (b equiv.) and H_2O (c equiv.) in solvent (0.25 M). Reactions were stirred at 90 °C for 16 h before aq. workup. Conversion was determined by HPLC with the use of caffeine as an internal standard.

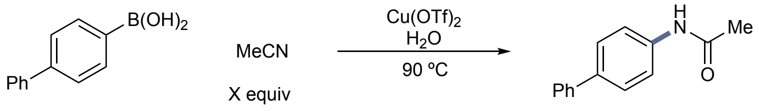

Entry	Solvent	$Cu(OTf)_2$	MeCN	H_2O	Yield
**1**	DMSO	3 equiv.	10 equiv.	3 equiv.	0%
**2**	DMF	3 equiv.	10 equiv.	3 equiv.	0%
**3**	CH_2Cl_2	3 equiv.	10 equiv.	3 equiv.	47%
**4**	DCE	3 equiv.	10 equiv.	3 equiv.	48%
**5**	THF	3 equiv.	10 equiv.	3 equiv.	74%
**6**	PhMe	3 equiv.	10 equiv.	3 equiv.	82%
**7**	PhMe	3 equiv.	3 equiv.	3 equiv.	38%
**8**	PhMe	3 equiv.	5 equiv.	3 equiv.	59%
**10**	PhMe	2 equiv.	10 equiv.	3 equiv.	33%
**11**	PhMe	4 equiv.	10 equiv.	3 equiv.	67%
**12**	**PhMe**	**4 equiv.**	**10 equiv.**	**2 equiv.**	**89%**
**13**	PhMe	4 equiv.	10 equiv.	1 equiv.	47%

Table S9: Attempts to render process catalytic with a terminal oxidant

Reactions carried out according to General Procedure A using 4-biphenylboronic acid (50 mg, 0.25 mmol, 1.0 equiv.), $Cu(OTf)_2$ (18 mg, 0.05 mmol, 20 mol%), and H_2O (13.0 µL, 0.75 mmol, 3.0 equiv.) and **oxidant** (5 equiv.) in MeCN (1 mL, 0.25 M) and stirred at 90 °C for X h before aq. workup. Conversion was determined by HPLC with the use of caffeine as an internal standard.

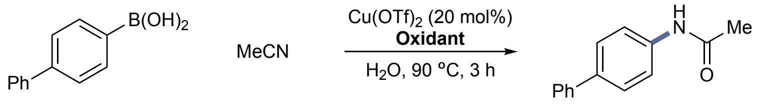

Entry	Oxidant	Time	Yield
**1**	Air	3 h	0%
**2**	O_2	3 h	0%
**3**	MnO_2	3 h	0%
**4**	MnO_2	18 h	0%
**5**	TBHP	3 h	0%
**6**	TBHP	18 h	0%

Table S10: Screening of the optimal conditions for heterocycle *N*-arylation

Reactions carried out according to General Procedure C using 4-biphenylboronic acid (50 mg, 0.25 mmol, 1.0 equiv.), $Cu(OTf)_2$ (a equiv.), 4-dimethylaminopyridine (b equiv.), and H_2O (c equiv.) in PhMe (0.25 M) and stirred at X °C for 16 h before filtration. Conversion was determined by ^1H NMR with the use of dibenzylether as an internal standard.

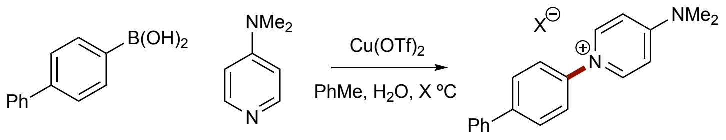

Entry	T(°C)	$Cu(OTf)_2$ (equiv.)	DMAP (equiv.)	H_2O (equiv.)	Yield (NMR)
1	50	3	10	0	5%
2	70	3	10	0	10%
3	90	2	10	0	2%
4	90	3	10	0	24%
5	90	4	10	0	39%
6	90	4	2	0	6%
7	90	4	3	0	27%
8	90	4	4	0	29%
9	90	4	5	0	34%
10	90	3	5	0	50%
11	90	4	5	1	37%
12	90	3	5	2	24%
13	90	3	5	3	27%
14[a]	90	3	5	0	66%
15[b]	90	3	5	0	70%
**16 [b,c]**	**90**	**3**	**5**	**0**	**89%**
17[b-d]	90	3	5	0	84%

[a]Recrystallized boronic acid; [b]Using 4-tolylboronic acid; [c]With prestirring of boronic acid and $Cu(OTf)_2$ in toluene for >1 h; [d] 0.125 M.

Table S11: Screening of alternate copper sources

Reactions carried out according to General Procedure C using 4-biphenylboronic acid (50 mg, 0.25 mmol, 1.0 equiv.), Cu Source (3 equiv.), and 4-dimethylaminopyridine (5 equiv.) in PhMe (0.25 M) and stirred at 90 °C for 16 h before filtration. Conversion was determined by ^{1}H NMR with the use of dibenzylether as an internal standard.

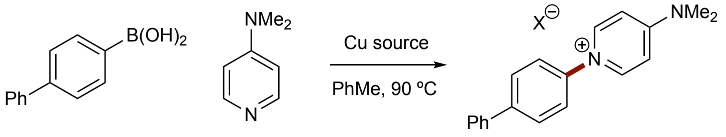

Entry	Cu source	Conversion
**1**	$CuCl_2$	0%
**2**	CuF_2	0%
**3**	$CuSO_4$	0%
**4**	$Cu(OAc)_2$	0%
**5**	$[Cu(MeCN)_4][BF_4]$	0%
**6**	$Cu(OTf)_2$	70%

Scheme S15: Attempts to employ conditions of You and coworkers in amide formation – $Cu(OAc)_2$.

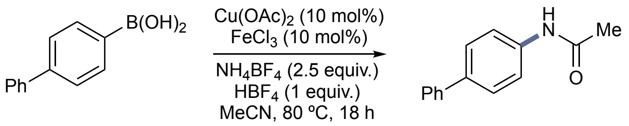

Procedure adapted from *Chem. Commun.* **2014**, *50*, 3941.

An oven dried 5 mL microwave vial was charged with 4-biphenylboronic acid (50 mg, 0.25 mmol, 1.0 equiv.), $Cu(OAc)_2$ (4.5 mg, 0.025 mmol, 10 mol%), $FeCl_3$ (6.8 mg, 0.025 mmol, 10 mol%), and NH_4BF_4 (65.5 mg, 0.625 mmol, 2.5 equiv.). The vial was then sealed under air before adding MeCN (1 mL, 0.25 M) and HBF_4 (44 µL, 0.25 mmol, 1 equiv.). The reaction mixture was then heated to 80 ºC for 18 h. The reaction was allowed to cool to room temperature, diluted with EtOAc (10 mL), washed with brine, and the organics were dried (Na_2SO_4) and concentrated under

vacuum. ^{1}H NMR analysis of the crude mixture showed no product, only clean biphenyl (the product of complete protodeboronation).

Scheme S16: Attempts to employ conditions of You and coworkers in amide formation – Cu(OTf)$_2$.

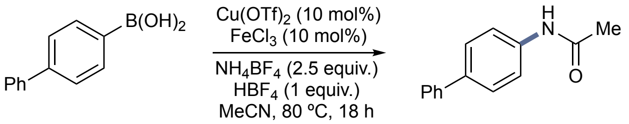

Procedure adapted from *Chem. Commun.* **2014**, *50*, 3941.

An oven dried 5 mL microwave vial was charged with 4-biphenylboronic acid (50 mg, 0.25 mmol, 1.0 equiv.), Cu(OTf)$_2$ (9.0 mg, 0.025 mmol, 10 mol%), $FeCl_3$ (6.8 mg, 0.025 mmol, 10 mol%), and NH_4BF_4 (65.5 mg, 0.625 mmol, 2.5 equiv.). The vial was then sealed under air before adding MeCN (1 mL, 0.25 M) and HBF_4 (44 µL, 0.25 mmol, 1 equiv.). The reaction mixture was then heated to 80 ºC for 18 h. The reaction was allowed to cool to room temperature, diluted with EtOAc (10 mL), washed with brine, and the organics were dried (Na_2SO_4) and concentrated under vacuum. ^{1}H NMR analysis of the crude mixture showed no product, only clean biphenyl (the product of complete protodeboronation).

Scheme S17: Attempts to employ conditions of You and coworkers in pyridinium formation.

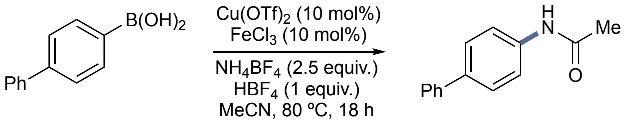

Procedure adapted from *Chem. Commun.* **2014**, *50*, 3941.

An oven dried 5 mL microwave vial was charged with 4-dimethylaminopyridine (30.5 mg, 0.25 mmol, 1 equiv.), 4-biphenylboronic acid (74.3 mg, 0.375 mmol, 1.5 equiv.), Cu(OTf)$_2$ (9.0 mg, 0.025 mmol, 10 mol%), $FeCl_3$ (6.8 mg, 0.025 mmol, 10 mol%), and NH_4BF_4 (65.5 mg, 0.625 mmol, 2.5 equiv.). The vial was then sealed under air before adding DMF (1 mL, 0.25 M) and HBF_4 (44 µL, 0.25 mmol, 1 equiv.). The reaction mixture was then heated to 80 ºC for 18 h. The reaction was allowed to cool to room temperature, diluted with EtOAc (10 mL) and dry loaded on

silica gel. Purification by flash chromatography (silica gel, 2–5% MeOH/DCM) afforded the desired product as a white solid in 10% yield.

6. Experimental Procedures and Characterization Data

Amide Substrate Scope (Scheme 7a)

***N*-([1,1'-biphenyl]-4-yl)acetamide, 3a**

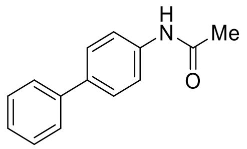

Prepared according to General Procedure A using [1,1'-biphenyl]-4-ylboronic acid (49.5 mg, 0.25 mmol, 1.0 equiv.), $Cu(OTf)_2$ (270 mg, 0.75 mmol, 3.0 equiv.), H_2O (13.0 µL, 0.75 mmol, 3.0 equiv.), and MeCN (1 mL, 0.25 M). The reaction mixture was subjected to the purification outlined in the General Procedure (silica gel, 0-33% EtOAc/petroleum ether) to afford the desired product as an off-white solid. (49.5 mg, 94%).

υ_{max} (solid): 3296, 3190, 2921, 2850, 1656, 1545, 834, 764, 738, 697 cm^{-1}.

^{1}H NMR (500 MHz, $CDCl_3$): δ 7.57 (m, 7H), 7.42 (dd, *J* = 7.6, 7.3 Hz, 2H), 7.33 (t, *J* = 7.3 Hz, 1H), 2.20 (s, 3H).

^{13}C NMR (126 MHz, $CDCl_3$): δ 168.6, 140.5, 137.2, 137.2, 128.8, 127.6, 127.1, 126.9, 120.3, 24.6.

The spectral data were consistent with those previously reported in the literature.[2]

***N*-(4-(methylsulfonyl)phenyl)acetamide, 24**

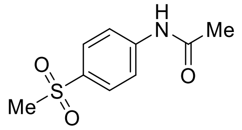

Prepared according to General Procedure A using (4-(methylsulfonyl)phenyl)boronic acid (50 mg, 0.25 mmol, 1.0 equiv.), $Cu(OTf)_2$ (270 mg, 0.75 mmol, 3.0 equiv.), H_2O (13 µL, 0.75 mmol, 3.0 equiv.), and MeCN (1 mL, 0.25 M). The reaction mixture was subjected to the purification outlined in the General Procedure (silica gel, 0-33% EtOAc/petroleum ether) to afford the desired product as an off-white solid (28 mg, 53%).

υ_{max} (solid): 3330, 2919, 2850, 1688, 1591, 1532, 1316, 1145, 965, 773 cm^{-1}.

^{1}H NMR (500 MHz, DMSO-d_6) δ 10.38 (br. s, 1H), 7.87 – 7.77 (m, 4H), 3.15 (s, 3H), 2.09 (s, 3H).

^{13}C NMR (126 MHz, DMSO-d_6) δ 169.2, 143.8, 134.4, 128.3, 118.6, 43.9, 24.2.

The spectral data were consistent with those previously reported in the literature.[3]

***N*-(4-(trifluoromethyl)phenyl)acetamide, 25**

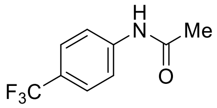

Prepared according to General Procedure A using (4-(trifluoromethyl)phenyl)boronic acid (47 mg, 0.25 mmol, 1.0 equiv.), $Cu(OTf)_2$ (270 mg, 0.75 mmol, 3.0 equiv.), H_2O (13 µL, 0.75 mmol, 3.0 equiv.), and MeCN (1 mL, 0.25 M). The reaction mixture was subjected to the purification outlined in the General Procedure (silica gel, 0-33% EtOAc/petroleum ether) to afford the desired product as an off white solid (35.4 mg, 70%).

υ_{max} (solid): 3321, 2923, 2473, 2447, 1665, 1391, 1114, 832 cm^{-1}.

^{1}H NMR (400 MHz, CD_3OD): δ 7.74 (d, J = 8.5 Hz, 2H), 7.58 (d, J = 8.5 Hz, 2H), 2.15 (s, 3H). (NH not observed).

^{13}C NMR (101 MHz, CD_3OD): δ 172.0, 143.5, 127.0 (q, $^3J_{CF}$ = 3.8 Hz), 126.5 (q, $^2J_{CF}$ = 32.5 Hz), 125.7 (q, $^1J_{CF}$ = 270.4 Hz), 120.6, 24.0.

^{19}F NMR (376 MHz, $CDCl_3$): δ –63.57.

The spectral data were consistent with those previously reported in the literature.[4]

***N*-(4-fluorophenyl)acetamide, 26**

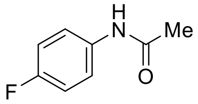

Prepared according to General Procedure A using (4-fluorophenyl)boronic acid (35 mg, 0.25 mmol, 1.0 equiv.), $Cu(OTf)_2$ (270 mg, 0.75 mmol, 3 equiv.), H_2O (13 µL, 0.75 mmol, 3.0 equiv.), and MeCN (1 mL, 0.25 M). The reaction mixture was subjected to the purification outlined in the General Procedure (silica gel, 0-33% EtOAc/petroleum ether) to afford the desired product as an off-white solid (36 mg, 94%).

υ_{max} (solid): 3287, 3264, 3220, 3147, 3069, 2958, 2922, 2850, 1660, 1508, 836 cm^{-1}.

^{1}H NMR (400 MHz, $CDCl_3$): δ 7.65 (br. s, 1H), 7.52 – 7.42 (m, 2H), 7.01 (app t, J = 8.7 Hz, 2H), 2.17 (s, 3H).

^{13}C NMR (101 MHz, $CDCl_3$): δ 168.1, 158.9 (d, $^1J_{CF}$ = 243.5 Hz), 133.4, 121.4 (d, $^3J_{CF}$ = 7.8 Hz), 115.1 (d, $^2J_{CF}$ = 22.5 Hz), 23.8.

^{19}F NMR (376 MHz, $CDCl_3$): δ –117.99.

The spectral data were consistent with those previously reported in the literature.[4]

***N*-(*o*-tolyl)acetamide, 27**

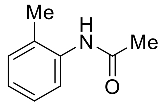

Prepared according to General Procedure A using 2-tolylboronic acid (34 mg, 0.25 mmol, 1.0 equiv.), $Cu(OTf)_2$ (270 mg, 0.75 mmol, 3.0 equiv.), H_2O (13 µL, 0.75 mmol, 3.0 equiv.), and MeCN (1.0 mL, 0.25 M). The reaction mixture was subjected to the purification outlined in the General Procedure (silica gel, 0-33% EtOAc/petroleum ether) to afford the desired product as an off white solid (14 mg, 38%).

υ_{max} (solid): 3285, 2852, 1649, 1587, 1526, 1370,1271, 756, 715, 701 cm^{-1}.

^{1}H NMR (500 MHz, $CDCl_3$): δ 7.71 (d, *J* = 7.5 Hz, 1H), 7.22 – 7.16 (m, 2H), 7.07 (m, 2H), 2.24 (s, 3H), 2.18 (s, 3H).

^{13}C NMR (126 MHz, $CDCl_3$): δ 168.3, 135.5, 130.3, 129.4, 126.6, 125.2, 123.5, 24.1, 17.6.

The spectral data were consistent with those previously reported in the literature.[4]

***N*-(3,5-dichlorophenyl)acetamide, 28**

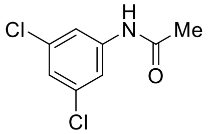

Prepared according to General Procedure A using (3,5-dichlorophenyl)boronic acid (64 mg, 0.25 mmol, 1.0 equiv.), $Cu(OTf)_2$ (270 mg, 0.75 mmol, 3.0 equiv.), H_2O (13.0 µL, 0.75 mmol, 3.0 equiv.), and MeCN (1 mL, 0.25 M). The reaction mixture was subjected to the purification outlined in the General Procedure (silica gel, 0-33% EtOAc/petroleum ether) to afford the desired product as light brown solid (41 mg, 81%).

υ_{max} (solid): 3290, 3253, 2956, 2924, 2852, 1666, 1593, 1554, 1446, 807 cm^{-1}.

^{1}H NMR (500 MHz, $CDCl_3$): δ 7.47 (s, 2H), 7.09 (s, 1H), 2.18 (s, 3H). (NH not observed)

^{13}C NMR (126 MHz, $CDCl_3$): δ 168.5, 139.8, 135.4, 124.4, 118.1, 24.8.

The spectral data were consistent with those previously reported in the literature.[5]

***N*-(naphthalen-2-yl)acetamide, 29**

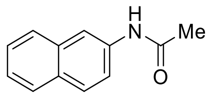

Prepared according to General Procedure A using naphthalen-2-ylboronic acid (43 mg, 0.25 mmol, 1.0 equiv.), $Cu(OTf)_2$ (270 mg, 0.75 mmol, 3.0 equiv.), H_2O (13.0 μL, 0.75 mmol, 3.0 equiv.), and MeCN (1 mL, 0.25 M). The reaction mixture was subjected to the purification outlined in the General Procedure (silica gel, 0-33% EtOAc/petroleum ether) to afford the desired product as light brown solid (23 mg, 50%).

υ_{max} (solid): 3304, 3054, 2924, 1666, 1556, 1372 cm^{-1}.

^{1}H NMR (500 MHz, $CDCl_3$): δ 8.18 (s, 1H), 7.79 – 7.75 (m, 3H), 7.52 (br. s, 1H), 7.47 – 7.43 (m, 2H), 7.40 (t, J = 7.4 Hz, 1H), 2.23 (s, 3H).

^{13}C NMR (126 MHz, $CDCl_3$): δ 168.7, 135.5, 134.0, 130.8, 128.9, 127.8, 127.7, 126.6, 125.2, 120.0, 116.8, 24.8.

The spectral data were consistent with those previously reported in the literature.[6]

***N*-(4-methoxyphenyl)acetamide, 30**

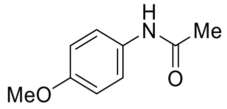

Prepared according to General Procedure A using (4-methoxyphenyl)boronic acid (38 mg, 0.25 mmol, 1.0 equiv.), $Cu(OTf)_2$ (270 mg, 0.75 mmol, 3.0 equiv.), H_2O (13 μL, 0.75 mmol, 3.0 equiv.), and MeCN (1 mL, 0.25 M). The reaction mixture was subjected to the purification outlined in the General Procedure (silica gel, 0-33% EtOAc/petroleum ether) to afford the desired product as an off-white solid (11.7 mg, 28%).

υ_{max} (solid): 3295, 3191, 2953, 2922, 2851, 1661, 1510, 1246, 832 cm^{-1}.

^{1}H NMR (500 MHz, $CDCl_3$): δ 7.38 (d, J = 8.9 Hz, 2H), 7.11 (s, 1H), 6.85 (d, J = 8.9 Hz, 2H), 3.79 (s, 3H), 2.15 (s, 3H).

^{13}C NMR (126 MHz, $CDCl_3$): δ 168.3, 156.6, 131.1, 122.1, 114.3, 55.6, 24.5.

The spectral data were consistent with those previously reported in the literature.[4]

***N*-(naphthalen-1-yl)acetamide, 31**

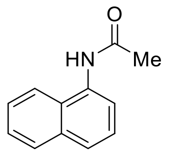

Prepared according to General Procedure A using naphthalen-1-ylboronic acid (43 mg, 0.25 mmol, 1.0 equiv.), $Cu(OTf)_2$ (270 mg, 0.75 mmol, 3.0 equiv.), H_2O (13.0 μL, 0.75 mmol, 3.0 equiv.), and MeCN (1 mL, 0.25 M). The reaction mixture was subjected to the purification outlined in the General Procedure (silica gel, 0-33% EtOAc/petroleum ether) to afford the desired product as light brown solid (20 mg, 43%).

υ_{max} (solid): 3249, 3049, 2955, 2922, 2851, 1663, 1536, 1505, 778 cm^{-1}.

^{1}H NMR (500 MHz, $CDCl_3$): δ 7.89 – 7.82 (m, 3H), 7.70 (d, *J* = 8.0 Hz, 1H), 7.58 (br. s, 1H), 7.55 – 7.43 (m, 3H), 2.30 (s, 3H).

^{13}C NMR (126 MHz, $CDCl_3$): δ 169.1, 134.3, 132.4, 128.9, 127.5, 126.4, 126.2, 126.1, 125.9, 121.6, 120.9, 24.4.

The spectral data were consistent with those previously reported in the literature.[7]

_N_-([1,1'-biphenyl]-4-yl)-2-phenylacetamide, 32

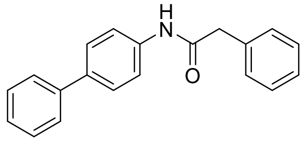

Prepared according to General Procedure B using [1,1'-biphenyl]-4-ylboronic acid (49.5 mg, 0.25 mmol, 1.0 equiv.), 2-phenylacetonitrile (288 μL, 2.5 mmol, 10.0 equiv.), $Cu(OTf)_2$ (360 mg, 1.0 mmol, 4.0 equiv.), H_2O (9 μL, 0.50 mmol, 2.0 equiv.), and PhMe (1 mL, 0.25 M). The reaction mixture was subjected to the purification outlined in the General Procedure (silica gel, 0-33% EtOAc/petroleum ether) to afford the desired product as a white solid (53 mg, 74%).

υ_{max} (solid): 3279, 3181, 3049, 1654, 1532, 764, 730, 719, 697 cm^{-1}.

^{1}H NMR (500 MHz, $CDCl_3$): δ 7.58 – 7.47 (m, 6H), 7.42 (m, 4H), 7.38 – 7.30 (m, 4H), 7.28 (s, 1H), 3.76 (s, 2H).

^{13}C NMR (126 MHz, $CDCl_3$): δ 169.3, 140.6, 137.5, 137.1, 134.6, 129.7, 129.4, 128.9, 127.8, 127.7, 127.3, 127.0, 120.3, 45.0.

HRMS (ESI): exact mass calculated for $[M+H]^+$ ($C_{20}H_{18}ON$) requires *m/z* 288.1383, found *m/z* 288.1385.

***N*-([1,1'-biphenyl]-4-yl)acrylamide, 33**

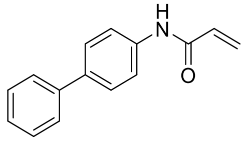

Prepared according to General Procedure B using [1,1'-biphenyl]-4-ylboronic acid (49.5 mg, 0.25 mmol, 1.0 equiv.) acrylonitrile (164 µL, 2.5 mmol, 10 equiv.), $Cu(OTf)_2$ (360 mg, 1.0 mmol, 4.0 equiv.), H_2O (9 µL, 0.50 mmol, 2.0 equiv.), and PhMe (1 mL, 0.25 M). The reaction mixture was subjected to the purification outlined in the General Procedure (silica gel, 0-33% EtOAc/petroleum ether) to afford the desired product as an off-white solid (46 mg, 82%).

υ_{max} (solid): 3303, 3033, 2949, 2923, 1665, 1542, 1410, 763 cm^{-1}.

^{1}H NMR (500 MHz, $CDCl_3$): δ 7.77 (s, 1H), 7.67 (d, *J* = 7.6 Hz, 2H), 7.56 (m, 4H), 7.42 (dd, *J* = 7.6, 7.3 Hz, 2H), 7.33 (t, *J* = 7.3 Hz, 1H), 6.46 (d, *J* = 16.8 Hz, 1H), 6.31 (dd, *J* = 16.8, 10.2 Hz, 1H), 5.77 (d, *J* = 10.2 Hz, 1H).

^{13}C NMR (126 MHz, $CDCl_3$): δ 163.9, 140.5, 137.5, 137.2, 131.3, 128.9, 128.0, 127.8, 127.3, 127.0, 120.6.

The spectral data were consistent with those previously reported in the literature.[8]

***N*-([1,1'-biphenyl]-4-yl)isobutyramide, 34**

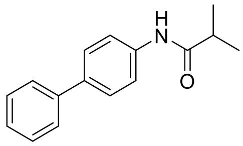

Prepared according to General Procedure B using [1,1'-biphenyl]-4-ylboronic acid (49.5 mg, 0.25 mmol, 1.0 equiv.) isobutyronitrile (225 µL, 2.5 mmol, 10 equiv.), $Cu(OTf)_2$ (360 mg, 1.0 mmol, 4.0 equiv.), H_2O (9 µL, 0.50 mmol, 2.0 equiv.), and PhMe (1 mL, 0.25 M). The reaction mixture was subjected to the purification outlined in the General Procedure (silica gel, 0-33% EtOAc/petroleum ether) to afford the desired product as a light brown solid (49.6 mg, 83%).

υ_{max} (solid): 3339, 2922, 2850, 1593, 1550, 1299, 1145, 840 cm^{-1}.

^{1}H NMR (400 MHz, $CDCl_3$): δ 7.62 (d, *J* = 8.6 Hz, 2H), 7.59 – 7.52 (m, 4H), 7.45 – 7.37 (m, 3H), 7.35 – 7.29 (m, 1H), 2.55 (hept, *J* = 6.9 Hz, 1H), 1.28 (d, *J* = 6.9 Hz, 6H)

^{13}**C NMR** (101 MHz, $CDCl_3$): δ 175.5, 140.6, 137.5, 137.1, 128.9, 127.7, 127.2, 126.9, 120.3, 36.8, 19.8.

HRMS (ESI): exact mass calculated for $[M+H]^+$ ($C_{16}H_{18}ON$) requires *m/z* 240.1383, found *m/z* 240.1381.

***N*-([1,1'-biphenyl]-4-yl)furan-3-carboxamide, 35**

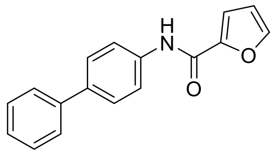

Prepared according to General Procedure B using [1,1'-biphenyl]-4-ylboronic acid (49.5 mg, 0.25 mmol, 1.0 equiv.), furan-2-carbonitrile (233 mg, 2.5 mmol, 10 equiv.), $Cu(OTf)_2$ (360 mg, 1.0 mmol, 4.0 equiv.), H_2O (9 µL, 0.50 mmol, 2.0 equiv.), and PhMe (1 mL, 0.25 M). The reaction mixture was subjected to the purification outlined in the General Procedure (silica gel, 0-33% EtOAc/petroleum ether) to afford the desired product as a white solid (33.5 mg, 51%).

υ_{max} (solid): 3358, 3097, 1668, 1520, 763, 746 cm^{-1}.

^{1}**H NMR** (500 MHz, $CDCl_3$): δ 8.09 (s, 1H), 7.65 (d, *J* = 8.5 Hz, 2H), 7.50 (m, 4H), 7.42 (s, 1H), 7.34 (t, *J* = 7.6 Hz, 2H), 7.24 (t, *J* = 7.3 Hz, 1H), 7.16 (d, *J* = 3.3 Hz, 1H), 6.47 (dd, *J* = 3.3, 1.6 Hz, 1H).

^{13}**C NMR** (126 MHz, $CDCl_3$): δ 156.2, 147.9, 144.4, 140.6, 137.5, 136.8, 128.9, 127.8, 127.3, 127.0, 120.4, 115.4, 112.8.

HRMS (ESI): exact mass calculated for $[M+H]^+$ ($C_{17}H_{14}O_2N$) requires *m/z* 264.1019, found *m/z* 264.1020.

***N*-([1,1'-biphenyl]-4-yl)-2-fluoro-4-methoxybenzamide, 36**

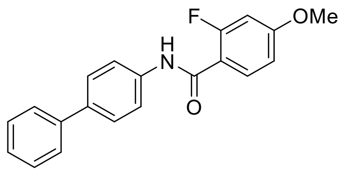

Prepared according to General Procedure B using [1,1'-biphenyl]-4-ylboronic acid (49.5 mg, 0.25 mmol, 1.0 equiv.), 2-fluoro-4-methoxybenzonitrile (382 mg, 2.5 mmol, 10 equiv.), $Cu(OTf)_2$ (360 mg, 1.0 mmol, 4.0 equiv.), H_2O (9 µL, 0.50 mmol, 2.0 equiv.), and PhMe (1 mL, 0.25 M). The reaction mixture was subjected to the purification outlined in the General

Procedure (silica gel, 0-33% EtOAc/petroleum ether) to afford the desired product as a white solid (50.6 mg, 63%).

υ_{max} (solid): 3310, 3003, 2964, 2927, 2836, 1652, 1529, 1497, 763 cm^{-1}.

^{1}H NMR (500 MHz, $CDCl_3$): δ 8.59 (d, J = 16.6 Hz, 1H), 7.75 (d, J = 8.5 Hz, 2H), 7.67 (dd, J = 6.1, 3.2 Hz, 1H), 7.65 – 7.57 (m, 4H), 7.45 (t, J = 7.7 Hz, 2H), 7.35 (t, J = 7.4 Hz, 1H), 7.10 (dd, J = 11.2, 9.1 Hz, 1H), 7.07 – 7.02 (m, 1H), 3.86 (s, 3H).

^{13}C NMR (126 MHz, $CDCl_3$): δ 161.3 (d, $^{3}J_{CF}$ = 3.8 Hz), 156.4, 155.0 (d, $^{1}J_{CF}$ = 238.9 Hz), 140.6, 137.8, 137.1, 128.9, 127.8, 127.3, 127.0, 121.6 (d, $^{2}J_{CF}$ = 12.8 Hz), 120.9, 120.6 (d, $^{3}J_{CF}$ = 9.4 Hz), 117.2 (d, $^{2}J_{CF}$ = 27.6 Hz), 114.9, 56.1.

^{19}F NMR (471 MHz, $CDCl_3$): δ –123.41.

HRMS (ESI): exact mass calculated for $[M+H]^+$ ($C_{20}H_{17}O_2NF$) requires *m/z* 322.1238, found *m/z* 322.1239.

***N*-([1,1'-biphenyl]-4-yl)-3-bromo-4-methylbenzamide, 37**

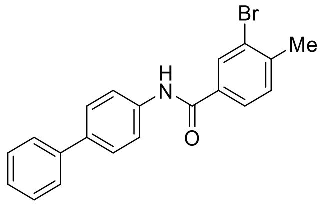

Prepared according to General Procedure B using [1,1'-biphenyl]-4-ylboronic acid (49.5 mg, 0.25 mmol, 1.0 equiv.) 3-bromo-4-methylbenzonitrile (487 mg, 2.5 mmol, 10 equiv.), $Cu(OTf)_2$ (360 mg, 1.0 mmol, 4.0 equiv.), H_2O (9 μL, 0.50 mmol, 2.0 equiv.), and PhMe (1 mL, 0.25 M). The reaction mixture was subjected to the purification outlined in the General Procedure (silica gel, 0-33% EtOAc/petroleum ether, 0-33%) to afford the desired product as a white solid (67.5 mg, 74%).

υ_{max} (solid): 3331, 2959, 2918, 2849, 1641, 1527, 763 cm^{-1}.

^{1}H NMR (400 MHz, DMSO): δ 10.36 (s, 1H), 8.21 (s, 1H), 7.94 – 7.85 (m, 3H), 7.72 – 7.64 (m, 4H), 7.53 (d, J = 7.9 Hz, 1H), 7.45 (t, J = 7.6 Hz, 2H), 7.34 (t, J = 7.3 Hz, 1H), 2.43 (s, 3H).

^{13}C NMR (101 MHz, DMSO): δ 163.8, 141.2, 139.7, 138.4, 135.4, 134.3, 131.1, 131.1, 128.9, 127.1, 127.1, 126.8, 126.3, 124.1, 120.7, 22.4.

HRMS (ESI): exact mass calculated for $[M+H]^+$ ($C_{20}H_{17}ONBr$) requires *m/z* 366.0488, found *m/z* 366.0490.

***N*-([1,1'-biphenyl]-4-yl)-2-(2-methoxyphenyl)acetamide, 38**

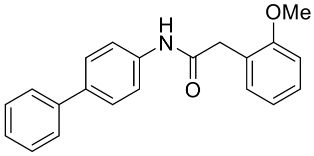

Prepared according to General Procedure B using [1,1'-biphenyl]-4-ylboronic acid (49.5 mg, 0.25 mmol, 1.0 equiv.) 2-(2-methoxyphenyl)acetonitrile (367 mg, 2.5 mmol, 10 equiv.), $Cu(OTf)_2$ (360 mg, 1.0 mmol, 4.0 equiv.), H_2O (9 µL, 0.50 mmol, 2.0 equiv.), and PhMe (1 mL, 0.25 M). The reaction mixture was subjected to the purification outlined in the General Procedure (silica gel, 0-33% EtOAc/petroleum ether) to afford the desired product as a white solid (66 mg, 87%).

υ_{max} (solid): 3277, 3028, 1658, 1524, 1245, 766, 751, 697 cm^{-1}.

^{1}H NMR (400 MHz, DMSO): δ 10.14 (s, 1H), 7.70 (d, J = 8.7 Hz, 2H), 7.66 – 7.59 (m, 4H), 7.44 (dd, J = 10.5, 4.9 Hz, 2H), 7.35 – 7.29 (m, 1H), 7.28 – 7.21 (m, 2H), 6.98 (d, J = 7.9 Hz, 1H), 6.91 (td, J = 7.4, 1.0 Hz, 1H), 3.78 (s, 3H), 3.65 (s, 2H).

^{13}C NMR (101 MHz, $CDCl_3$): δ 169.6, 157.1, 140.7, 137.6, 136.9, 131.5, 129.2, 128.9, 127.6, 127.1, 126.9, 123.4, 121.5, 120.0, 111.0, 55.7, 40.3.

HRMS (ESI): exact mass calculated for $[M+H]^+$ ($C_{21}H_{20}O_2N$) requires *m/z* 318.1489, found *m/z* 318.1490.

N-Aryl heterocycle Substrate Scope (Scheme 7b)

4-Ph-Ph-DMAP$^+$OTf$^-$, 20

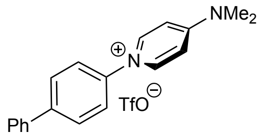

Prepared according to General Procedure C using [1,1'-biphenyl]-4-ylboronic acid (99 mg, 0.5 mmol, 1.0 equiv.), Cu(OTf)$_2$ (253 mg, 1.5 mmol, 3.0 equiv.), and 4-dimethylaminopyridine (305 mg, 2.5 mmol, 5.0 equiv.) in PhMe (2 mL, 0.25 M). NMR assay of the reaction mixture after 16 h showed 70% conversion to product. The reaction mixture was subjected to the purification outlined in the General Procedure (basic alumina, DCM:MeCN, 1:1) to afford the desired product as a white solid. (31.2 mg, 43%). Single crystals of **10** were grown by slow diffusion of diethylether into a DCM solution of this material (CSD 1971680).

υ_{max} (solid): 3264, 3055, 3036, 1647, 1595, 1568, 1519, 1483, 1448, 1404, 1269, 1244, 1217, 1198, 1150, 1028, 999, 833, 756, 687, 635 cm^{-1}.

^{1}H NMR (400 MHz, CDCl$_3$): δ 8.26 (d, J = 7.8 Hz, 2H), 7.72 (d, J = 8.7 Hz, 2H), 7.65 – 7.51 (m, 4H), 7.50 – 7.42 (m, 2H), 7.39 (t, J = 7.3 Hz, 1H), 7.12 (d, J = 7.9 Hz, 2H), 3.27 (s, 6H).

^{13}C NMR (126 MHz, CDCl$_3$): δ 156.6, 142.9, 141.0, 140.8, 138.9, 129.2, 129.1, 128.5, 127.2, 123.5, 121.0 (q, $^1J_{CF}$ = 320 Hz), 108.9, 40.6.

^{19}F NMR (471 MHz, CDCl$_3$): δ –78.17.

HRMS (ESI): exact mass calculated for [M]$^+$ ($C_{19}H_{19}N_2$) requires *m/z* 275.1548, found *m/z* 275.1532.

[4-F$_3$CO-Ph-DMAP]$^+$[OTf]$^-$, 39

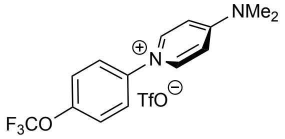

Prepared according to General Procedure C using (4-trifluoromethoxyphenyl)boronic acid (103 mg, 0.5 mmol, 1.0 equiv.), Cu(OTf)$_2$ (543 mg, 1.5 mmol, 3.0 equiv.), and 4-dimethylaminopyridine (305 mg, 2.5 mmol, 5.0 equiv.) in PhMe (2 mL, 0.25 M). NMR assay of the reaction mixture after 16 h showed 90% conversion to product. The reaction mixture was

subjected to the purification outlined in the General Procedure (basic alumina, DCM:MeCN, 1:1) to afford the desired product as a white solid. (61 mg, 41%)

υ$_{max}$ (solid): 3265, 3142, 3105, 3092, 3073, 1645, 1564, 1445, 1402, 1271, 1242, 1215, 1147, 1026, 997, 943, 833, 798, 754, 633, 573 cm^{-1}.

^{1}H NMR (400 MHz, CD_3OD): δ 8.40 (d, J = 7.9 Hz, 2H), 7.98 – 7.67 (m, 2H), 7.67 – 7.35 (m, 2H), 7.23 – 7.01 (m, 2H), 3.35 (s, 6H).

^{13}C NMR (126 MHz, CD_3OD): δ 158.2, 151.0, 142.4, 142.2, 126.7, 123.9, 121.8 (q, $^{1}J_{CF}$ = 257.0 Hz), 121.8 (q, $^{1}J_{CF}$ = 318.4 Hz), 109.2, 40.6.

^{19}F NMR (471 MHz, $CDCl_3$): δ –59.57, –80.02.

HRMS (ESI): exact mass calculated for $[M]^+$ ($C_{14}H_{14}ON_2F_3$) requires *m/z* 283.1058, found *m/z* 283.1046.

[2-Me-Ph-DMAP]$^+$[OTf]$^-$, 40

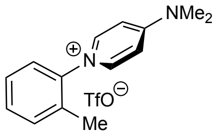

Prepared according to General Procedure C using 2-tolylboronic acid (34.0 mg, 0.25 mmol, 1.0 equiv.), $Cu(OTf)_2$ (270 mg, 0.75 mmol, 3.0 equiv.), and 4-dimethylaminopyridine (153 mg, 1.25 mmol, 5.0 equiv.) in PhMe (1 mL, 0.25 M). NMR assay of the reaction mixture after 16 h showed 40% conversion to product. The reaction mixture was subjected to the purification outlined in the General Procedure (basic alumina, DCM:MeCN, 1:1) to afford the desired product as a pale yellow solid. (11.8 mg, 13%)

υ$_{max}$ (solid): 2970, 2920, 2901, 1645, 1576, 1518, 1489, 1445, 1406, 1346, 1261, 1219, 1190, 1144, 1030 941, 835, 820, 773, 754, 721, 637, 573 cm^{-1}.

^{1}H NMR (400 MHz, $CDCl_3$): δ 7.94 (d, J = 7.8 Hz, 2H), 7.47 (td, J = 7.5, 1.3 Hz, 1H), 7.38 (t, J = 8.1 Hz, 2H), 7.29 (d, J = 7.7 Hz, 1H), 7.17 (d, J = 7.8 Hz, 2H), 3.36 (s, 6H), 2.19 (s, 3H).

^{13}C NMR (126 MHz, $CDCl_3$): δ 156.8, 142.2, 141.1, 133.3, 132.3, 131.0, 128.1, 126.1, 120.9 (app. d, $^{1}J_{CF}$ = 319.8 Hz), 108.7, 40.8, 17.4.

^{19}F NMR (471 MHz, $CDCl_3$): δ –78.27.

HRMS (ESI): exact mass calculated for $[M]^+$ ($C_{14}H_{17}N_2$) requires *m/z* 213.1391, found *m/z* 213.1385.

[4-F-Ph-DMAP]$^+$[OTf]$^-$, 41

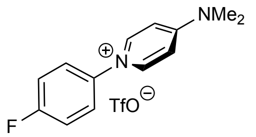

Prepared according to General Procedure C using (4-fluorophenyl)boronic acid (34.0 mg, 0.25 mmol, 1.0 equiv.), $Cu(OTf)_2$ (270 mg, 0.75 mmol, 3.0 equiv.), and 4-dimethylaminopyridine (153 mg, 1.25 mmol, 5.0 equiv.) in PhMe (1 mL, 0.25 M). NMR assay of the reaction mixture after 16 h showed 77% conversion to product. The reaction mixture was subjected to the purification outlined in the General Procedure (basic alumina, DCM:MeCN, 1:1) to afford the desired product as an off-white solid. (38.4 mg, 42%)

υ_{max} (solid): 1645, 1564, 1508, 1444, 1406, 1265, 1221, 1142, 1032, 1020, 1009, 845, 825, 810, 631, 573 cm^{-1}.z

^{1}H NMR (400 MHz, $CDCl_3$): δ 8.21 (d, J = 7.4 Hz, 2H), 7.52 (dd, J = 8.9, 4.4 Hz, 2H), 7.22 (dd, J = 8.9, 7.8 Hz, 2H), 7.09 (d, J = 7.4 Hz, 2H), 3.29 (s, 6H).

^{13}C NMR (126 MHz, CD_3OD): δ 164.36 (d, $^1J_{CF}$ = 249.1 Hz), 158.1, 142.5, 140.0 (d, $^4J_{CF}$ = 3.1 Hz), 127.0 (d, $^3J_{CF}$ = 9.0 Hz), 121.8 (q, $^1J_{CF}$ = 318.6 Hz), 118.2 (d, $^2J_{CF}$ = 23.7 Hz), 109.1, 40.6.

^{19}F NMR (471 MHz, $CDCl_3$): δ –79.99, –113.14.

HRMS (ESI): exact mass calculated for [M]$^+$ ($C_{13}H_{14}FN_2$) requires *m/z* 217.1141, found *m/z* 217.1129.

[3,5-Cl_2-Ph-DMAP]$^+$[OTf]$^-$, 42

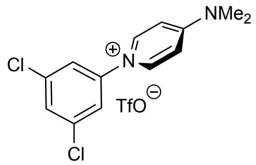

Prepared according to General Procedure C using (3,5-dichlorophenyl)boronic acid (48.0 mg, 0.25 mmol, 1.0 equiv.), $Cu(OTf)_2$ (270 mg, 0.75 mmol, 3.0 equiv.), and 4-dimethylaminopyridine (153 mg, 1.25 mmol, 5.0 equiv.) in PhMe (1 mL, 0.25 M). NMR assay of the reaction mixture after 16 h showed 58% conversion to product. The reaction mixture was subjected to the purification outlined in the General Procedure (basic alumina, DCM:MeCN, 1:1) to afford the desired product as a yellow solid. (17.6 mg, 17%)

υ_{max} (solid): 3059, 1647, 1578, 1431, 1406, 1271, 1254, 1219, 1161, 1121, 1107, 1065, 1025, 893, 873, 824, 804, 739, 689, 635, 571 cm^{-1}.

^{1}H NMR (400 MHz, $CDCl_3$): δ 8.29 – 8.11 (m, 2H), 7.50 (t, *J* = 1.7 Hz, 1H), 7.46 (d, *J* = 1.7 Hz, 2H), 7.22 – 7.00 (m, 2H), 3.34 (s, 6H).

^{13}C NMR (126 MHz, $CDCl_3$): δ 157.0, 143.1, 140.8, 137.1, 130.4, 122.3, 120.9 (q, $^1J_{CF}$ = 320 Hz), 109.2, 40.9.

^{19}F NMR (471 MHz, $CDCl_3$): δ –78.25.

HRMS (ESI): exact mass calculated for $[M]^+$ ($C_{13}H_{13}N_2Cl_2$) requires *m/z* 267.0455, found *m/z* 267.0440.

$[4\text{-Me-Ph-DMAP}]^+[OTf]^-$, 21

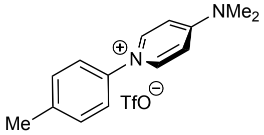

Prepared according to General Procedure C using 4-tolylboronic acid (34.0 mg, 0.25 mmol, 1.0 equiv.), $Cu(OTf)_2$ (270 mg, 0.75 mmol, 3.0 equiv.), and 4-dimethylaminopyridine (153 mg, 1.25 mmol, 5.0 equiv.) in PhMe (1 mL, 0.25 M). NMR assay of the reaction mixture after 16 h showed 89% conversion to product. The reaction mixture was subjected to the purification outlined in the General Procedure (basic alumina, DCM:MeCN, 1:1) to afford the desired product as a white solid. (26.6 mg, 46%)

υ_{max} (solid): 1649, 1387, 1263, 1236, 1222, 1215, 1195, 1157, 1020, 812 cm^{-1}.

^{1}H NMR (400 MHz, CD_3OD): δ 8.64 – 8.10 (m, 2H), 7.53 – 7.35 (m, 4H), 7.21 – 6.98 (m, 2H), 3.33 (s, 6H), 2.45 (s, 3H).

^{13}C NMR (126 MHz, $CDCl_3$): δ 156.7, 141.1, 140.6, 139.6, 131.2, 123.0, 120.9 (q, $^1J_{CF}$ = 320.5 Hz), 108.9, 40.7, 21.2.

^{19}F NMR (471 MHz, $CDCl_3$): δ –78.24.

HRMS (ESI): exact mass calculated for $[M]^+$ ($C_{14}H_{17}N_2$) requires *m/z* 213.1391, found *m/z* 213.1379.

$[5\text{-benzofuranyl-DMAP}]^+[OTf]^-$, 43

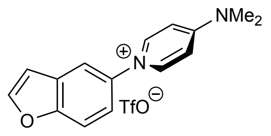

Prepared according to General Procedure C using (5-benzofuranyl)boronic acid (40.5 mg, 0.25 mmol, 1.0 equiv.), $Cu(OTf)_2$ (270 mg, 0.75 mmol, 3.0 equiv.), and 4-dimethylaminopyridine (153 mg, 1.25 mmol, 5.0 equiv.) in PhMe (1 mL, 0.25 M). NMR assay of the reaction mixture after 16 h showed 65% conversion to product. The reaction mixture was subjected to the purification outlined in the General Procedure (basic alumina, DCM:MeCN, 1:1) to afford the desired product as an off-white solid. (32.5 mg, 34%)

υ_{max} (solid): 1645, 1576, 1558, 1464, 1258, 1223, 1161, 1134, 1119, 1026, 1013, 945, 868, 831, 806, 779, 766, 754, 741, 635, 571 cm^{-1}.

^{1}H NMR (400 MHz, $CDCl_3$): δ 8.37 – 8.13 (m, 2H), 7.74 (dd, *J* = 7.2, 2.3 Hz, 2H), 7.58 (dt, *J* = 8.8, 0.7 Hz, 1H), 7.34 (dd, *J* = 8.7, 2.4 Hz, 1H), 7.17 – 7.02 (m, 2H), 6.86 (dd, *J* = 2.2, 0.9 Hz, 1H), 3.27 (s, 6H).

^{13}C NMR (126 MHz, $CDCl_3$): δ 156.5, 154.7, 147.9, 141.7, 137.5, 129.1, 120.9 (q $^1J_{CF}$ = 320.7 Hz), 119.6, 116.6, 113.1, 108.7, 107.3, 40.6.

^{19}F NMR (471 MHz, $CDCl_3$): δ –78.25.

HRMS (ESI): exact mass calculated for $[M]^+$ ($C_{15}H_{15}ON_2$) requires *m/z* 239.1184, found *m/z* 239.1171.

[3-thiophenyl-DMAP]$^+$[OTf]$^-$, 44

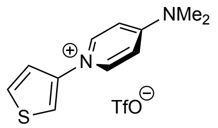

Prepared according to General Procedure C using (3-thiophenyl)boronic acid (30 mg, 0.25 mmol, 1.0 equiv.), $Cu(OTf)_2$ (270 mg, 0.75 mmol, 3.0 equiv.), and 4-dimethylaminopyridine (153 mg, 1.25 mmol, 5.0 equiv.) in PhMe (1 mL, 0.25 M). NMR assay of the reaction mixture after 16 h showed 20% conversion to product. The reaction mixture was subjected to the purification outlined in the General Procedure (basic alumina, DCM:MeCN, 1:1) to afford the desired product as an off-white solid. (5.4 mg, 6%)

υ_{max} (solid): 3059, 2359, 2344, 2330, 1647, 1578, 1431, 1408, 1256, 1219, 1159, 1130, 1121, 1107, 1065, 1028, 893, 874, 825, 806, 754, 739, 689, 635, 571 cm^{-1}.

^{1}**H NMR** (400 MHz, $CDCl_3$): δ 8.28 (d, *J* = 7.4 Hz, 2H), 7.67 (d, *J* = 2.8 Hz, 1H), 7.55 (d, *J* = 4.5 Hz, 1H), 7.30 (d, *J* = 5.7 Hz, 1H), 7.12 (d, *J* = 7.4 Hz, 2H), 3.35 (s, 6H).
^{13}**C NMR** (126 MHz, $CDCl_3$): δ 156.7, 140.9, 139.8, 129.1, 121.7, 118.6, 108.8, 40.8. (CF_3 from $^{-}$OTf counteranion not observed).
^{19}**F NMR** (471 MHz, $CDCl_3$): δ –78.25.
HRMS (ESI): exact mass calculated for $[M]^+$ ($C_{11}H_{13}SN_2$) requires *m/z* 205.07994, found *m/z* 205.0788.

[1-octyl-DMAP]$^+$[OTf]$^-$, 45

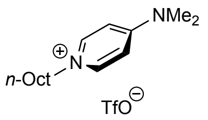

Prepared according to General Procedure C using *n*-octylboronic acid (40 mg, 0.25 mmol, 1.0 equiv.), $Cu(OTf)_2$ (270 mg, 0.75 mmol, 3.0 equiv.), and 4-dimethylaminopyridine (153 mg, 1.25 mmol, 5.0 equiv.) in PhMe (1 mL, 0.25 M). NMR assay of the reaction mixture after 16 h showed 33% conversion to product. The reaction mixture was subjected to the purification outlined in the General Procedure (basic alumina, DCM:MeCN, 1:1) to afford the desired product as an off-white solid. (12.5 mg, 13%)
υ_{max} (solid): 1653, 1570, 1558, 1260, 1225, 1157, 1030, 638, 573 cm^{-1}.
^{1}**H NMR** (400 MHz, $CDCl_3$): δ 8.08 (d, *J* = 7.4 Hz, 2H), 6.92 (d, *J* = 7.3 Hz, 2H), 4.14 (t, *J* = 7.3 Hz, 2H), 1.91 – 1.66 (m, 2H), 1.43 – 1.18 (m, 12H), 0.85 (t, *J* = 6.9 Hz, 3H).
^{13}**C NMR** (126 MHz, $CDCl_3$): δ 156.3, 142.0, 120.8 (q, $^{1}J_{CF}$ = 320.5 Hz), 108.3, 58.5, 40.3, 31.7, 31.0, 29.0, 29.0, 26.1, 22.6, 14.1.
^{19}**F NMR (**471 MHz, $CDCl_3$): δ –78.28.
HRMS (ESI): exact mass calculated for $[M]^+$ ($C_{15}H_{27}N_2$) requires *m/z* 235.2174, found *m/z* 235.2163.

[4-Tol-(3-F-Py)]$^+$[OTf]$^-$, 46

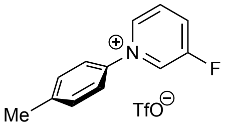

Prepared according to General Procedure C using 4-tolylboronic acid (34 mg, 0.25 mmol, 1.0 equiv.), $Cu(OTf)_2$ (271 mg, 0.75 mmol, 3.0 equiv.), and 3-fluoropyridine (121 μL, 1.25 mmol,

5.0 equiv.) in PhMe (1 mL, 0.25 M). NMR assay of the reaction mixture after 16 h showed 44% conversion to product. The reaction mixture was subjected to the purification outlined in the General Procedure (basic alumina, DCM:MeCN, 1:1) to afford the desired product as an off-white solid. (9 mg, 11%)

υ_{max} (solid): 1647, 1603, 1576, 1558, 1491, 1456, 1292, 1260, 1225, 1157, 1030, 895, 824, 799, 756, 735, 721, 679, 637, 575 cm^{-1}.

^{1}H NMR (400 MHz, CD_3OD): δ 9.55 (tq, J = 2.2, 1.1 Hz, 1H), 9.27 – 9.04 (m, 1H), 8.69 (ddd, J = 9.5, 7.3, 2.5 Hz, 1H), 8.31 (dt, J = 8.9, 5.7 Hz, 1H), 7.92 – 7.65 (m, 2H), 7.56 (d, J = 8.1 Hz, 2H), 2.51 (s, 3H).

^{13}C NMR (126 MHz, CD_3OD): δ 162.1 (d, $^1J_{CF}$ = 255.7 Hz), 144.3, 143.1, 141.79, 136.5 (d, $^2J_{CF}$ = 39.4 Hz), 134.8 (d, $^2J_{CF}$ = 18.7 Hz), 132.2, 130.8 (d, $^3J_{CF}$ = 7.9 Hz), 125.2, 121.8 (app. d, $^1J_{CF}$ = 318.6 Hz), 21.2.

^{19}F NMR (471 MHz, CD_3OD): δ –80.12, –116.82.

HRMS (ESI): exact mass calculated for $[M]^+$ ($C_{12}H_{11}NF$) requires *m/z* 188.0875, found *m/z* 188.0860.

$[4\text{-Tol-Py}]^+[OTf]^-$, 47

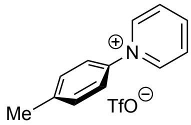

Prepared according to General Procedure C using 4-tolylboronic acid (68 mg, 0.5 mmol, 1.0 equiv.), $Cu(OTf)_2$ (543 mg, 1.5 mmol, 3.0 equiv.), and pyridine (202 µL, 2.5 mmol, 5.0 equiv.) in PhMe (2 mL, 0.25 M). NMR assay of the reaction mixture after 16 h showed 44% conversion to product. The reaction mixture was subjected to the purification outlined in the General Procedure (basic alumina, DCM:MeCN, 1:1) to afford the desired product as an off white solid. (19.1 mg, 20%)

^{1}H NMR (400 MHz, CD_3OD): δ 9.38 – 9.11 (m, 2H), 8.93 – 8.59 (m, 1H), 8.28 (dd, J = 8.0, 6.6 Hz, 2H), 7.82 – 7.66 (m, 2H), 7.66 – 7.36 (m, 2H), 2.53 (s, 3H).

^{13}C NMR (126 MHz, CD_3OD): δ 147.6, 146.0, 143.8, 132.2, 129.5, 125.2, 121.8 (app. d, $^1J_{CF}$ = 318.5 Hz), 21.2.

The spectral data were consistent with those previously reported in the literature.[9]

[4-Tol-(4-OMe-Py)]$^+$[OTf]$^-$, 48

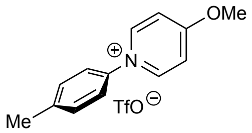

Prepared according to General Procedure C using 4-tolylboronic acid (34 mg, 0.25 mmol, 1.0 equiv.), $Cu(OTf)_2$ (270 mg, 0.75 mmol, 3.0 equiv.) and 4-methoxypyridine (127 µL, 1.25 mmol, 5.0 equiv.) in PhMe (1 mL, 0.25 M). NMR assay of the reaction mixture after 16 h showed 50% conversion to product. The reaction mixture was subjected to the purification outlined in the General Procedure (basic alumina, DCM:MeCN, 1:1) to afford the desired product as an off white solid. (11.4 mg, 13%)

υ_{max} (solid): 1638, 1508, 1321, 1252, 1225, 1204, 1159, 1028, 1015, 999, 822, 638, 573 cm^{-1}.

^{1}H NMR (400 MHz, $CDCl_3$): δ 8.74 (dd, J = 7.8, 2.7 Hz, 2H), 7.68 (dd, J = 7.9, 2.5 Hz, 2H), 7.49 (dd, J = 8.5, 2.5 Hz, 2H), 7.40 (d, J = 8.2 Hz, 2H), 4.20 (s, 3H), 2.45 (s, 3H).

^{13}C NMR (126 MHz, $CDCl_3$): δ 172.1, 145.4, 142.1, 139.6, 131.5, 123.5, 120.8 (app. d, $^{1}J_{CF}$ = 320.2 Hz), 114.7, 58.8, 21.3.

^{19}F NMR (471 MHz, $CDCl_3$): δ –78.32.

HRMS (ESI): exact mass calculated for $[M]^+$ ($C_{13}H_{14}NO$) requires *m/z* 200.1075, found *m/z* 200.1066.

[4-Tol-(3-Me-quinoline)]$^+$[OTf]$^-$, 49

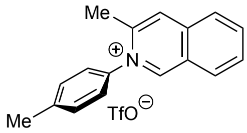

Prepared according to General Procedure C using 4-tolylboronic acid (34 mg, 0.25 mmol, 1.0 equiv.), $Cu(OTf)_2$ (370 mg, 0.75 mmol, 3.0 equiv.) and 3-methylisoquinoline (179 mg, 1.25 mmol, 5.0 equiv.) in PhMe (1 mL, 0.25 M). The reaction mixture was subjected to the purification outlined in the General Procedure (basic alumina, DCM:MeCN, 0-50%) to afford the desired product as an off-white solid. (27 mg, 28%)

υ_{max} (solid): 1647, 1508, 1435, 1396, 1261, 1157, 1030.

^{1}H NMR (400 MHz, CD_3CN): 9.57 (s, 1H), 8.41 – 8.35 (m, 2H), 8.25 – 8.21 (m, 2H), 8.00 (ddd, J = 8.3, 4.9, 3.1 Hz, 1H), 7.54 (dt, J = 8.0, 0.7 Hz, 2H), 7.51 – 7.46 (m, 2H), 2.53 (s, 3H), 2.51 (s, 3H).

^{13}**C NMR** (126 MHz, CD_3CN): δ 152.0, 146.0, 143.0, 140.2, 139.7, 138.8, 132.0, 131.7, 131.1, 127.6, 127.3, 127.1, 126.6, 122.1 (q, $^{1}J_{CF}$ = 320.8 Hz), 21.3, 20.9.
^{19}**F NMR** (377 MHz, $CDCl_3$): δ –78.44.
HRMS (ESI): exact mass calculated for $[M]^+$ ($C_{17}H_{16}N$) requires *m/z* 234.1277, found *m/z* 234.1268.

[4-Tol-(N-Me-Imid)]$^+$[OTf]$^-$, 50

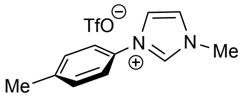

Prepared according to General Procedure C using 4-tolylboronic acid (34 mg, 0.25 mmol, 1.0 equiv.), $Cu(OTf)_2$ (270 mg, 0.75 mmol, 3.0 equiv.) and *N*-methylimidazole (100 µL, 1.25 mmol, 5.0 equiv.) in PhMe (1 mL, 0.25 M). NMR assay of the reaction mixture after 16 h showed 87% conversion to product. The reaction mixture was subjected to the purification outlined in the General Procedure (basic alumina, DCM:MeCN, 1:1) to afford the desired product as an off white solid. (20.8 mg, 26%)
$υ_{max}$ (solid): 1578, 1558, 1514, 1258, 1225, 1148, 1076, 1030, 816, 754, 637, 615, 572 cm^{-1}.
^{1}**H NMR** (400 MHz, $CDCl_3$): δ 9.45 – 9.29 (m, 1H), 7.59 (dt, *J* = 14.5, 1.9 Hz, 2H), 7.53 – 7.40 (m, 2H), 7.40 – 7.28 (m, 2H), 4.05 (s, 3H), 2.38 (s, 3H).
^{13}**C NMR** (126 MHz, $CDCl_3$): δ 140.9, 135.3, 132.1, 131.1, 124.7, 121.8, 121.3, 120.7 (q, $^{1}J_{CF}$ = 320.0 Hz), 36.8, 21.2.
^{19}**F NMR** (471 MHz, $CDCl_3$): δ –78.52.
HRMS (NSI): exact mass calculated for $[M]^+$ ($C_{11}H_{13}N_2$) requires *m/z* 173.1078, found *m/z* 173.1068.

***N*,*N*(α)-Phthalimide-N(im)-methyl-L-histidine methyl ester (Phth-His(Me)-OMe), S5**

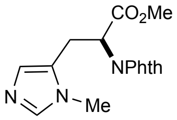

Prepared by an adapted literature procedure[10] from Phth-His-OMe.[11]

Phth-His-OMe (2.73 g, 9.12 mmol, 1 equiv.) was dissolved in MeCN under N_2 atmosphere. Et_3N (1.53 mL, 1.2 equiv) and a solution of TsCl in MeCN (1.91 g in 10 mL, 1.1 equiv) were

successively added dropwise, and the mixture was stirred for 8 h. MeOTf (3 mL, 1.5 equiv) was then added. After 24 h, piperidine (2.3 mL, 2.6 equiv) was added, and the solution was heated at 80 °C overnight. After cooling to room temperature, DCM (100 mL) was added, and the mixture was washed twice with 2 M aq. NaOH (50 mL). The combined aqueous phases were then re-extracted with DCM (100 mL). The combined organic phases were dried (Na_2SO_4) and concentrated under reduced pressure to yield an orange oil which was triturated with diethyl ether to yield **10** as a beige powder (975.2 mg, 31%).

υ_{max} (solid): 1773, 1746, 1709, 1503, 1466, 1433, 1387, 1356, 1339, 1290, 1265, 1246, 1225, 122, 1167, 1109, 1098, 1063, 1022, 1005, 974, 918, 868, 841, 827, 789, 745, 719, 667, 640 cm^{-1}.

^{1}H NMR (500 MHz, $CDCl_3$): δ 7.80 (dd, *J* = 5.4, 3.1 Hz, 2H), 7.71 (dd, *J* = 5.5, 3.0 Hz, 2H), 7.32 (s, 1H), 6.70 (s, 1H), 5.10 (dd, *J* = 11.7, 4.8 Hz, 1H), 3.79 (s, 3H), 3.65 (dd, *J* = 15.9, 11.7 Hz, 1H), 3.64 (s, 3H), 3.44 (dd, *J* = 15.9, 4.8 Hz 1H).

^{13}C NMR (126 MHz, $CDCl_3$): δ 169.0, 167.5, 138.6, 134.5, 131.6, 128.5, 126.7, 123.8, 53.3, 50.6, 31.5, 23.7.

HRMS (ESI): exact mass calculated for $[M+H]^+$ ($C_{16}H_{26}N_3O_4$) requires *m/z* 314.1141, found *m/z* 314.1129.

[4-Tol-(Phth-His(Me)-OMe)]$^+$[OTf]$^-$, 51

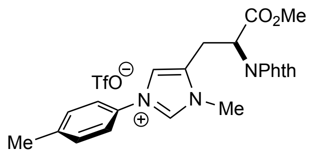

Prepared according to General Procedure C using 4-tolylboronic acid (34 mg, 0.25 mmol, 1.0 equiv.), $Cu(OTf)_2$ (370 mg, 0.75 mmol, 3.0 equiv.), and **S1** (vide infra, 392 mg, 1.25 mmol, 5.0 equiv.) in PhMe (1 mL, 0.25 M). NMR assay of the reaction mixture after 16 h showed 50% conversion to product. The reaction mixture was subjected to the purification outlined in the General Procedure (basic alumina, DCM:MeCN, 0-50%) to afford the desired product as a colourless solid. (29.3 mg, 29%)

υ_{max} (solid): 3136, 3074, 1773, 1746, 1713, 1560, 1516, 1470, 1431, 1383, 1362, 1279, 1252, 1223, 1196, 1155, 1107, 1088, 1030, 976, 910, 887, 868, 820, 789, 756, 723, 656, 637, 627, 573 cm^{-1}.

^{1}H NMR (400 MHz, $CDCl_3$): δ 9.52 (d, *J* = 1.7 Hz, 1H), 7.87 (dd, *J* = 5.5, 3.0 Hz, 2H), 7.78 (dd, *J* = 5.5, 3.0 Hz, 2H), 7.44 (d, *J* = 1.7 Hz, 1H), 7.39 (d, *J* = 8.4 Hz, 2H), 7.30 (d, *J* = 8.4 Hz, 2H), 5.21 (dd, *J* = 9.5, 5.4 Hz, 1H), 3.98 (s, 3H), 3.79 (s, 3H), 3.74 (ddd, *J* = 16.5, 5.4, 1.1 Hz, 1H), 3.55 (ddd, *J* = 16.5, 9.5, 1.1 Hz, 1H), 2.39 (s, 3H).

^{13}C NMR (126 MHz, $CDCl_3$): δ 167.9, 167.6, 140.9, 136.3, 135.0, 132.5, 132.1, 131.4, 131.2, 124.2, 121.8, 120.7 (q, $^1J_{CF}$ = 320.3 Hz), 118.6, 53.7, 49.4, 34.4, 24.3, 21.2.

^{19}F NMR (471 MHz, $CDCl_3$): δ –78.54.

HRMS (ESI): exact mass calculated for $[M]^+$ ($C_{23}H_{22}N_3O_4$) requires *m/z* 404.1610, found *m/z* 404.1596.

[4-Tol-(DBU)]$^+$[OTf]$^-$, 52 (Scheme 7c)

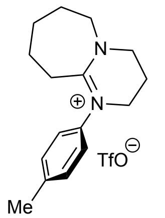

Prepared according to General Procedure C using 4-tolylboronic acid (68 mg, 0.5 mmol, 1.0 equiv.), $Cu(OTf)_2$ (540 mg, 1.5 mmol, 3.0 equiv.), and 1,8-diazabicyclo(5.4.0)undec-7-ene (374 µL, 2.5 mmol, 5.0 equiv.) in PhMe (2 mL, 0.25 M). NMR assay of the reaction mixture after 16 h showed 78% conversion to product. The reaction mixture was subjected to the purification outlined in the General Procedure (basic alumina, DCM:MeCN, 1:1) to afford the desired product as an off white solid. (55.7 mg, 28%)

υ_{max} (solid): 3275, 3148, 2984, 2938, 2889, 2866, 1734, 1647, 1628, 1593, 1508, 1474, 1449, 1325, 1242, 1221, 1207, 1148, 1105, 1026, 983, 829, 756, 690, 635, 573 cm^{-1}.

^{1}H NMR (400 MHz, $CDCl_3$): δ 7.22 (s, 4H), 3.73 (q, J = 5.8 Hz, 6H), 2.64 – 2.49 (m, 2H), 2.35 (s, 3H), 2.33 – 2.15 (m, 2H), 1.88 – 1.68 (m, 3H), 1.58 (m, 2H).

^{13}C NMR (126 MHz, $CDCl_3$): δ 167.0, 139.78, 139.77, 131.1, 126.4, 121.0 (q, $^1J_{CF}$ = 320.7 Hz), 55.9, 50.2, 49.4, 30.5, 28.7, 25.8, 23.1, 21.2, 20.1.

^{19}F NMR (471 MHz, $CDCl_3$): δ –78.21.

HRMS (ESI): exact mass calculated for $[M]^+$ ($C_{16}H_{23}N_2$) requires *m/z* 243.1861, found *m/z* 243.1848.

[4-Tol-(Tetramisole)]$^+$[OTf]$^-$, 53 (Scheme 7d)

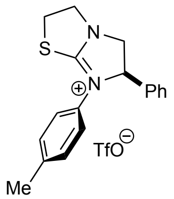

Prepared according to General Procedure C using 4-tolylboronic acid (34 mg, 0.25 mmol, 1.0 equiv.), $Cu(OTf)_2$ (270 mg, 0.75 mmol, 3.0 equiv.), and (–)-tetramisole (255 mg, 1.25 mmol, 5.0 equiv.) in PhMe (1 mL, 0.25 M). NMR assay of the reaction mixture after 16 h showed 54% conversion to product. The reaction mixture was subjected to the purification outlined in the

General Procedure (basic alumina, DCM:MeCN, 0-33%) to afford the desired product as pale yellow solid (39.1 mg, 35%)

υ_{max} (solid): 2972, 2957, 2922, 2901, 2887, 1591, 1578, 1516, 1497, 1456, 1337, 1306, 1258, 1223, 1152, 1030, 822, 766, 756, 735, 702, 669, 638, 573 cm^{-1}.

^{1}H NMR (400 MHz, $CDCl_3$): δ 7.45 – 7.31 (m, 5H), 7.09 (s, 4H), 6.13 (dd, *J* = 10.8, 8.4 Hz, 1H), 4.67 (app. t, *J* = 10.6 Hz, 1H), 4.34 – 4.17 (m, 1H), 4.14 – 4.02 (m, 3H), 3.95 (dd, *J* = 10.4, 8.4 Hz, 1H), 2.26 (s, 3H).

^{13}C NMR (126 MHz, $CDCl_3$): δ 176.3, 139.5, 135.6, 132.7, 130.6, 129.8, 129.4, 127.9, 123.9, 120.9 (q, $^1J_{CF}$ = 320 Hz), 74.0, 55.0, 48.8, 36.4, 21.1.

^{19}F NMR (471 MHz, $CDCl_3$): δ –78.14

HRMS (ESI): exact mass calculated for $[M]^+$ ($C_{18}H_{19}N_2S$) requires *m/z* 295.1269, found *m/z* 295.1257.

1-(2-methoxyphenyl)-1*H*-imidazole, 54

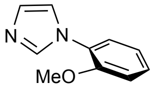

Prepared according to the procedure of Xie and coworkers.[12]

To a 250 mL round bottomed flask was added (2-methoxyphenyl)boronic acid (5.0 g, 33 mmol, 1.0 equiv.), imidazole (2.7 g, 39.6 mmol, 1.2 equiv.), CuCl (163 mg, 1.65 mmol, 5 mol%), and MeOH (132 mL, 0.25 M). The flask was fitted with a reflux condenser and heated to 70 ºC for 16 h. The reaction mixture was filtered through celite and concentrated under vacuum. The crude material was purified by column chromatography (silica gel, 70% EtOAc/petroleum ether) to afford the desired product as a colorless oil (750 mg, 13%).

^{1}H NMR (400 MHz, $CDCl_3$): δ 7.79 (br. s, 1H), 7.36 (ddd, *J* = 8.3, 7.5, 1.7 Hz, 1H), 7.29 (dd, *J* = 7.7, 1.7 Hz, 1H), 7.19 (d, *J* = 14.3 Hz, 2H), 7.09 – 7.01 (m, 2H), 3.85 (s, 3H).

The spectral data were consistent with those previously reported in the literature.[12]

[4-OCF_3-Ph-(2-OMe-Ph-imidazole)]$^+$[OTf]$^-$, 56 (Scheme 7e)

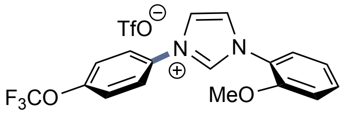

Prepared according to General Procedure C using (4-trifluoromethoxyphenyl)boronic acid (154 mg, 0.75 mmol, 1.0 equiv.), $Cu(OTf)_2$ (810 mg, 2.25 mmol, 3.0 equiv.), and imidazole **54** (653 mg, 3.75 mmol, 5.0 equiv.) in PhMe (3 mL, 0.25 M). The reaction mixture was cooled to room temperature and DCM was added (along with 1 equiv. dibenzyl ether as an internal standard). The suspension was filtered through fluted filter paper and dried under reduced pressure. NMR assay of the reaction mixture after 16 h showed 50% conversion to product. The reaction mixture was subjected to the purification outlined in the General Procedure (basic alumina, DCM:MeCN, 1:1) to afford the desired product as an off white solid (38 mg, 11%). Residue collected from the filter paper was dissolved in a small quantity of DCM and recrystallized by vapor diffusion with hexanes to afford single crystals of intermediate Cu complex **55** as a green solid, the structure of which was confirmed by X-ray analysis (CSD 1971681).

υ_{max} (solid): 1558, 1501, 1249, 1236, 1223, 1161, 1030, 761.
^{1}H NMR (500 MHz, $CDCl_3$): δ 9.66 (s, 1H), 7.98 (s, 1H), 7.89 (d, J = 7.9 Hz, 2H), 7.71 (s, 1H), 7.60 (d, J = 8.2 Hz, 1H), 7.48 (t, J = 8.0 Hz, 1H), 7.38 (d, J = 8.5 Hz, 2H), 7.10 (d, J = 8.4 Hz, 1H), 7.07 (t, J = 7.8 Hz, 1H), 3.89 (s, 3H).
^{13}C NMR (126 MHz, $CDCl_3$): δ 152.1, 150.4, 135.2, 132.6, 132.4, 125.9, 124.8, 124.4, 122.9, 120.7 (app. d, $^1J_{CF}$ = 320.1 Hz), 120.3 (app. d, $^1J_{CF}$ = 259.2 Hz), 112.8, 56.3.
^{19}F NMR (471 MHz, $CDCl_3$): δ –58.27, -78.55.
HRMS (ESI): exact mass calculated for $[M]^+$ ($C_{17}H_{14}N_2O_2F_3$) requires *m/z* 335.1002, found *m/z* 335.0990.

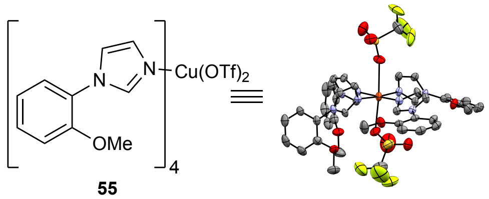

55

Tolvaptan intermediate, 57 (Scheme 7f)

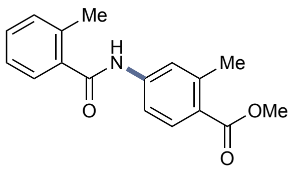

Prepared according to General Procedure B using (4-(methoxycarbonyl)-3-methylphenyl)boronic acid (48 mg, 0.25 mmol, 1.0 equiv.), $Cu(OTf)_2$ (360 mg, 1.0 mmol, 4.0 equiv.), and 2-methylbenzonitrile (0.3 mL, 2.5 mmol, 10.0 equiv.) and H_2O (9 μL, 0.5 mmol, 2.0 equiv.) in PhMe (1 mL, 0.25 M). The reaction mixture was subjected to the purification outlined in the General Procedure (silica gel, EtOAc/petroleum ether, 5-10%) to afford the desired product as white solid (38 mg, 54%).

υ_{max} (solid): 1713, 1651, 1582, 1520, 1435, 1269, 1242, 1146, 1086, 833.

^{1}H NMR (500 MHz, $CDCl_3$): δ 8.00 – 7.95 (m, 1H), 7.61 – 7.50 (m, 3H), 7.48 (dd, *J* = 7.6, 1.4 Hz, 1H), 7.38 (td, *J* = 7.5, 1.4 Hz, 1H), 7.30 – 7.25 (m, 2H), 3.88 (s, 3H), 2.63 (s, 3H), 2.51 (s, 3H).

^{13}C NMR (126 MHz, $CDCl_3$): δ 168.4, 167.5, 142.2, 141.4, 136.6, 136.1, 132.2, 131.4, 130.6, 126.7, 126.0, 125.0, 122.1, 116.6, 51.8, 22.2, 19.9.

HRMS (ESI): exact mass calculated for $[M+Na]^+$ ($C_{17}H_{17}NO_3Na$) requires *m/z* 306.1101, found *m/z* 306.1094.

[4-F-Ph-(Pyriproxyfen)]$^+$[OTf]$^-$, 58 (Scheme 7g)

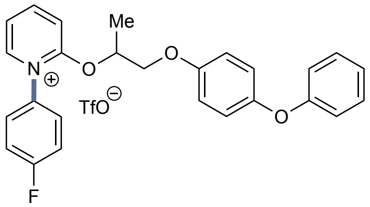

Prepared according to General Procedure C using 4-(fluoro)phenylboronic acid (14 mg, 0.1 mmol, 1.0 equiv.), $Cu(OTf)_2$ (108 mg, 0.3 mmol, 3.0 equiv.) and Pyriproxyfen (96 mg, 0.3 mmol, 3.0 equiv.) in PhMe (0.5 mL, 0.2 M). NMR assay of the reaction mixture after 16 h showed 27% conversion to product. The reaction mixture was subjected to the purification outlined in the General Procedure (basic alumina, DCM:MeCN, 1:1) to afford the desired product as a colorless oil (4 mg, 6%).

υ_{max} (solid): 2962, 2918, 2848, 1504, 1259, 1085, 1016, 798.

^{1}H NMR (400 MHz, $CDCl_3$): δ 8.55 (ddd, *J* = 9.2, 7.4, 1.9 Hz, 1H), 8.16 (dd, *J* = 6.4, 1.8 Hz, 1H), 8.00 (d, *J* = 9.0 Hz, 1H), 7.64 – 7.52 (m, 3H), 7.31 (dd, *J* = 8.6, 7.3 Hz, 2H), 7.22 (dd, *J* = 9.0, 7.7 Hz, 2H), 7.09 – 7.04 (m, 1H), 6.97 – 6.90 (m, 4H), 6.81 – 6.71 (m, 2H), 5.55 (td, *J* = 6.7, 2.6 Hz, 1H), 4.15 (dd, *J* = 11.0, 2.7 Hz, 1H), 4.00 (dd, *J* = 10.9, 7.2 Hz, 1H), 1.51 (d, *J* = 6.4 Hz, 3H).
^{13}C NMR (101 MHz, $CDCl_3$): δ 162.5, 159.2 (d, $^{1}J_{CF}$ = 213.5 Hz), 154.0, 151.3, 149.6, 142.6, 129.8, 128.3 (d, $^{3}J_{CF}$ = 9.1 Hz), 122.9, 120.9, 119.3, 118.0, 117.3 (d, $^{2}J_{CF}$ = 23.6 Hz), 115.7, 114.5, 80.9, 70.7, 29.8, 16.2. (CF_3 from $^{-}$OTf counteranion not observed).
^{19}F NMR (471 MHz, $CDCl_3$): δ –78.28, –107.70.
HRMS (ESI): exact mass calculated for $[M]^+$ ($C_{26}H_{23}FNO_3$) requires *m/z* 416.1656, found *m/z* 416.1647.

Additional Substrate Scope (Scheme S15)

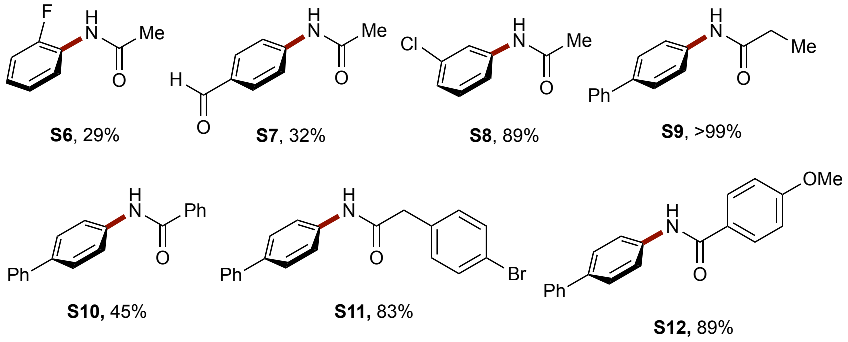

N-(2-fluorophenyl)acetamide, S6

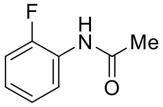

Prepared according to General Procedure A using (2-fluorophenyl)boronic acid (35 mg, 0.25 mmol, 1.0 equiv.), $Cu(OTf)_2$ (270 mg, 0.75 mmol, 3.0 equiv.), H_2O (13 µL, 0.75 mmol, 3.0 equiv.), and MeCN (1 mL, 0.25 M). The reaction mixture was subjected to the purification outlined in the General Procedure (silica gel, EtOAc/petroleum ether, 0-33%) to afford the desired product as an off white solid (11 mg, 29%).

υ_{max} (solid): 3331, 2922, 1697, 1593, 1296, 1145, 771 cm^{-1}.

^{1}H NMR (400 MHz, CD_3OD): δ 7.88 – 7.83 (m, 1H), 7.18 – 7.09 (m, 3H), 2.16 (s, 3H). (NH not observed)

^{13}C NMR (101 MHz, CD_3OD): δ 172.1, 155.8 (d, $^1J_{CF}$ = 245.4 Hz), 127.1 (d, $^2J_{CF}$ = 11.7 Hz), 126.9 (d, $^3J_{CF}$ = 7.7 Hz), 125.8, 125.2 (d, $^4J_{CF}$ = 3.3 Hz), 116.4 (d, $^2J_{CF}$ = 19.8 Hz), 23.4.

^{19}F NMR (376 MHz, CD_3OD): δ –127.94.

The spectral data were consistent with those previously reported in the literature.[13]

N-(4-formylphenyl)acetamide, S7

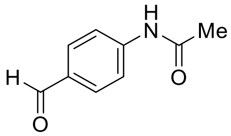

Prepared according to General Procedure A using (4-formylphenyl) boronic acid (37.5 mg, 0.25 mmol, 1.0 equiv.), $Cu(OTf)_2$ (270 mg, 0.75 mmol, 3.0 equiv.), H_2O (13 µL, 0.75 mmol, 3.0 equiv.), and MeCN (1 mL, 0.25 M). The reaction mixture was subjected to the purification

outlined in the General Procedure (silica gel, EtOAc/petroleum ether, 0-33%) to afford the desired product as an off white solid (13 mg, 32%).

υ_{max} (solid): 3307, 3263, 3192, 3121, 1690, 1677, 1600, 1535, 1271, 834 cm^{-1}.

^{1}H NMR (400 MHz, $CDCl_3$): δ 9.91 (s, 1H), 8.23 – 7.76 (m, 2H), 7.72 – 7.68 (m, 3H), 2.22 (s, 3H).

^{13}C NMR (101 MHz, $CDCl_3$): δ 191.5, 169.1, 144.0, 132.8, 131.6, 119.7, 25.3.

The spectral data were consistent with those previously reported in the literature.[14]

***N*-(3-chlorophenyl)acetamide, S8**

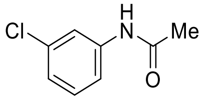

Prepared according to General Procedure A using (3-chlorophenyl) boronic acid (39 mg, 0.25 mmol, 1.0 equiv.), $Cu(OTf)_2$ (270 mg, 0.75 mmol, 3.0 equiv.), H_2O (13.0 µL, 0.75 mmol, 3.0 equiv.), and MeCN (1 mL, 0.25 M). The reaction mixture was subjected to the purification outlined in the General Procedure (silica gel, EtOAc/petroleum ether, 0-33%) to afford the desired product as white solid (37.6 mg, 89%).

υ_{max} (solid): 3298, 3185, 3119, 2924, 1667, 1593, 1537, 1483, 1422, 1405, 779 cm^{-1}.

^{1}H NMR (500 MHz, $CDCl_3$): δ 7.85 (s, 1H), 7.62 (s, 1H), 7.34 (d, J = 7.9 Hz, 1H), 7.20 (t, J = 7.9 Hz, 1H), 7.06 (d, J = 7.9 Hz, 1H), 2.16 (s, 3H).

^{13}C NMR (126 MHz, $CDCl_3$): δ 169.0, 139.2, 134.7, 130.1, 124.5, 120.2, 118.1, 24.6.

The spectral data were consistent with those previously reported in the literature.[4]

***N*-([1,1'-biphenyl]-4-yl)propionamide, S9**

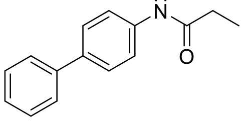

Prepared according to General Procedure B using [1,1'-biphenyl]-4-ylboronic acid (49.5 mg, 0.25 mmol, 1.0 equiv.), propiononitrile (174 µL, 2.5 mmol, 10 equiv.), $Cu(OTf)_2$ (360 mg, 1.0 mmol, 4.0 equiv.), H_2O (9 µL, 0.50 mmol, 2.0 equiv.), and PhMe (1 mL, 0.25 M). The reaction mixture was subjected to the purification outlined in the General Procedure (silica gel, EtOAc/petroleum ether, 0-33%) to afford the desired product as a light brown solid (60 mg, >99%).

υ_{max} **(solid):** 3277, 2970, 2922, 2849, 1655, 1536, 761 cm^{-1}.

^{1}H NMR (500 MHz, $CDCl_3$): δ 7.63 (s, 1H), 7.61 (d, *J* = 8.5 Hz, 2H), 7.55 (m, 4H), 7.42 (apt, *J* = 7.6 Hz, 2H), 7.33 (t, *J* = 7.3 Hz, 1H), 2.42 (q, *J* = 7.6 Hz, 2H), 1.26 (t, *J* = 7.6 Hz, 3H).

^{13}C NMR (126 MHz, $CDCl_3$): δ 172.4, 140.6, 137.5, 137.9, 128.9, 127.6, 127.2, 126.9, 120.4, 30.8, 9.8.

HRMS (NSI): exact mass calculated for $[M+H]^+$ ($C_{15}H_{16}ON$) requires *m/z* 226.1226, found *m/z* 226.1226.

***N*-([1,1'-biphenyl]-4-yl)benzamide, S10**

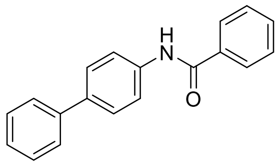

Prepared according to General Procedure B using [1,1'-biphenyl]-4-ylboronic acid (49.5 mg, 0.25 mmol, 1.0 equiv.), benzonitrile (256 μL, 2.5 mmol, 10 equiv.), $Cu(OTf)_2$ (360 mg, 1.0 mmol, 4.0 equiv.), H_2O (9 μL, 0.50 mmol, 2.0 equiv.), and PhMe (1 mL, 0.25 M). The reaction mixture was subjected to the purification outlined in the General Procedure (silica gel, EtOAc/petroleum ether, 0-33%) to afford the desired product as a colorless solid (31 mg, 45%).

υ_{max} **(solid):** 3344, 2953, 2922, 2851, 1654, 1490, 761 cm^{-1}.

^{1}H NMR (500 MHz, DMSO): δ 10.34 (s, 1H), 7.98 (d, *J* = 7.2 Hz, 2H), 7.90 (d, *J* = 8.6 Hz, 2H), 7.68 (m, 4H), 7.60 (d, *J* = 7.2 Hz, 1H), 7.55 (t, *J* = 7.4 Hz, 2H), 7.46 (t, *J* = 7.7 Hz, 2H), 7.34 (t, *J* = 7.3 Hz, 1H).

^{13}C NMR (126 MHz, DMSO): δ 165.6, 139.7, 138.7, 135.3, 134.9, 131.6, 128.9, 128.4, 127.7, 127.1, 126.8, 126.3, 120.6.

The spectral data were consistent with those previously reported in the literature.[15]

***N*-([1,1'-biphenyl]-4-yl)-2-(4-bromophenyl)acetamide, S11**

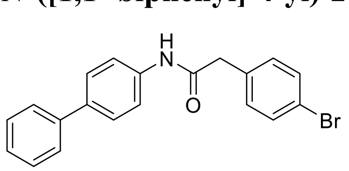

Prepared according to General Procedure B using [1,1'-biphenyl]-4-ylboronic acid (49.5 mg, 0.25 mmol, 1.0 equiv.), 2-(4-bromophenyl)acetonitrile (490 mg, 2.5 mmol, 10 equiv.), $Cu(OTf)_2$

(360 mg, 1.0 mmol, 4.0 equiv.), H_2O (9 μL, 0.50 mmol, 2.0 equiv.), and PhMe (1 mL, 0.25 M). The reaction mixture was subjected to the purification outlined in the General Procedure (silica gel, EtOAc/petroleum ether, 0-33%) to afford the desired product as a light brown solid (76 mg, 83%).

υ_{max} (solid): 3276, 2932, 2919, 1654, 1600, 1487, 834, 762 cm^{-1}.

^{1}H NMR (400 MHz, DMSO): δ 10.29 (s, 1H), 7.70 (d, *J* = 8.7 Hz, 2H), 7.65 – 7.58 (m, 4H), 7.52 (d, J = 8.4 Hz, 2H), 7.43 (t, *J* = 7.7 Hz, 2H), 7.37 – 7.27 (m, 3H), 3.67 (s, 2H).

^{13}C NMR (101 MHz, DMSO): δ 168.7, 139.7, 138.6, 135.4, 135.0, 131.4, 131.2, 128.9, 127.0, 126.9, 126.2, 119.8, 119.5, 42.5.

HRMS (NSI): exact mass calculated for $[M+H]^+$ ($C_{20}H_{17}ONBr$) requires *m/z* 366.0488, found *m/z* 366.0491.

***N*-([1,1'-biphenyl]-4-yl)-2-(4-methoxyphenyl)acetamide, S12**

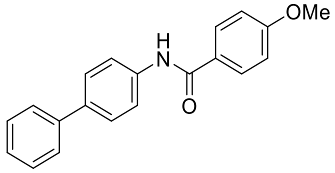

Prepared according to General Procedure B using [1,1'-biphenyl]-4-ylboronic acid (49.5 mg, 0.25 mmol, 1.0 equiv.), 4-methoxybenzonitrile (333 mg, 2.5 mmol, 10 equiv.), $Cu(OTf)_2$ (360 mg, 1.0 mmol, 4.0 equiv.), H_2O (9 μL, 0.50 mmol, 2.0 equiv.), and PhMe (1 mL, 0.25 M). The reaction mixture was subjected to the purification outlined in the General Procedure (silica gel, EtOAc/petroleum ether, 0-33%) to afford the desired product as a colorless solid (67 mg, 89%).

υ_{max} (solid): 3332, 2964, 2925, 1652, 1505, 834, 763 cm^{-1}.

^{1}H NMR (500 MHz, DMSO): δ 10.17 (s, 1H), 7.98 (d, *J* = 8.7 Hz, 2H), 7.88 (d, *J* = 8.5 Hz, 2H), 7.67 (m, 4H), 7.45 (dd, *J* = 7.7, 7.3 Hz, 2H), 7.33 (t, *J* = 7.3 Hz, 1H), 7.08 (d, *J* = 8.7 Hz, 2H), 3.85 (s, 3H).

^{13}C NMR (101 MHz, DMSO): δ 164.9, 161.9, 139.7, 138.9, 135.0, 129.6, 128.9, 127.0, 126.9, 126.7, 126.3, 120.6, 113.6, 55.4.

HRMS (NSI): exact mass calculated for $[M+H]^+$ ($C_{20}H_{18}O_2N$) requires *m/z* 304.1332, found *m/z* 304.1337.

7. X-ray Crystallography

For all structures: $Z = 4$. Experiments were carried out at 173 K with Mo $K\alpha$ radiation using a Rigaku XtaLAB P200K Diffractometer with multiscan absorption correction. H-atom parameters were constrained. Data was reduced using *CrystalClear*-SM Expert 2.1.[16] Structures were solved with ShelXT[17] and refined using ShelXL[18] within the Olex2 suite.[19] Figures for publication were produced using Mercury[20] and POV-ray[21] software packages.

Table S12. Crystallographic Experimental details

	10a/b	19	55	S1	S2
Crystal data					
Chemical formula	$C_{30}H_{40}CuF_6N_8O_6S_2$	$CF_3O_3S{\cdot}C_{19}H_{19}N_2$	$C_{42}H_{40}CuF_6N_8O_{10}S_2$	$C_8H_{16}CuF_6N_2O_6S_2$	$C_{14}H_{33}Cu_2F_6N_4O_7S_2{\cdot}CF_3O_3S$
M_r	850.37	424.43	1058.48	477.89	823.71
Space group	Orthorhombic, $P2_12_12_1$	Monoclinic, $P2_1/c$	Monoclinic, $P2_1/n$	Monoclinic, $I2/a$	Tetragonal, $P4_2/n$
a, b, c (Å)	11.6877 (2), 17.7725 (4), 18.5407 (3)	18.5990 (12), 7.1964 (4), 21.6252 (18)	15.0136 (19), 17.624 (2), 18.194 (2)	9.5844 (2), 9.6270 (2), 17.8387 (4)	13.0545 (1), 13.0545 (1), 36.4144 (5)
α, β, γ (°)	90, 90, 90	90, 131.803 (7), 90	90, 108.293 (3), 90	90, 91.111 (2), 90	90, 90, 90
V (Å^3)	3851.27 (13)	2157.6 (3)	4570.8 (9)	1645.65 (6)	6205.74 (13)
μ (mm^{-1})	4	4	4	4	8
Crystal size (mm)	0.75	0.20	0.66	1.68	1.68
Data collection					
T_{min}, T_{max}	0.693, 1.000	0.726, 1.000	0.908, 0.974	0.672, 1.000	0.814, 1.000
No. of measured, independent and observed reflections	23837, 7691, 7239 [$I > 2\sigma(I)$]	32690, 4845, 2949 [$I > 2\sigma(I)$]	55150, 8342, 6268 [$F^2 > 2.0\sigma(F^2)$]	10760, 1779, 1757 [$I > 2\sigma(I)$]	8056, 8056, 7515 [$I > 2\sigma(I)$]
R_{int}	0.019	0.052	0.166	0.014	?
(sin θ/λ)$_{max}$ (Å^{-1})	0.665	0.673	0.602	0.667	0.703
Refinement					
$R[F^2 > 2\sigma(F^2)]$, $wR(F^2)$, S	0.034, 0.112, 0.99	0.086, 0.278, 1.05	0.085, 0.244, 1.05	0.018, 0.049, 1.08	0.057, 0.156, 1.07
Reflections	7691	4845	8342	1779	8056
Parameters	486	264	626	116	474
Restraints	0	0	0	0	119
Δ〉$_{max}$, Δ〉$_{min}$ (e Å^{-3})	$w = 1/[\sigma^2(F_o^2) + (0.1P)^2]$ where $P = (F_o^2 + 2F_c^2)/3$	$w = 1/[\sigma^2(F_o^2) + (0.1492P)^2 + 1.7296P]$ where $P = (F_o^2 + 2F_c^2)/3$	$w = 1/[\sigma^2(F_o^2) + (0.1099P)^2 + 20.084P]$ where $P = (F_o^2 + 2F_c^2)/3$	$w = 1/[\sigma^2(F_o^2) + (0.0251P)^2 + 1.6537P]$ where $P = (F_o^2 + 2F_c^2)/3$	$w = 1/[\sigma^2(F_o^2) + (0.0826P)^2 + 12.0005P]$ where $P = (F_o^2 + 2F_c^2)/3$
Abs struct.	0.59, -0.48	0.93, -0.61	3.09, -1.77	0.34, -0.29	1.24, -1.36
Absolute structure parameter	Flack[22] x determined using 2875 quotients	–	–	–	–

	[(I+)-(I-)]/[(I+)+(I-)]				

8. References

1. J. Irangu, M. J. Ferguson, R. B. Jordan, *Inorg. Chem.* **2005**, *44*, 1619–1625.

2. L. Tang, Z.-L. Wang, H.-L. Wan, Y.-H. He, Z. Guan, *Org. Lett.* **2020***, 22,* 6182–6186.

3. D. Antonow, T. Marrafa, I. Dawood, T. M. R. Ahmed, Haque, D. E. Thurston, G. Zinzalla, *Chem. Comm.* **2010**, *46*, 2289–2291.

4. P. Debnath, M. Baeten, N. Lefèvre, S. Van Daele, B. U. W. Maes, *Adv. Synth. Catal.* **2015**, *357*, 197–209.

5. N. Vodnala, R. Gujjarappa, C. K. Hazra, D. Kaldhi, A. K. Kabi, U. Beifuss, C. C. Malakar, *Adv. Synth. Catal.* **2019**, *361*, 135–145.

6. Y. Gao, J. Liu, Z. Li, T. Guo, S. Xu, H. Zhu, F. Wei, S. Chen, H. Gebru, K. Guo, *J. Org. Chem.* **2018***, 83,* 2040−2049.

7. K. Hyodo, G. Hasegawa, N. Oishi, K. Kuroda, K. Uchida, *J. Org. Chem.* **2018**, *83*, 13080−13087.

8. V. J. Cee, L. P. Volak, Y. Chen, M. D. Bartberger, C. Tegley, T. Arvedson, J. McCarter, A. S. Tasker, C. Fotsch, *J. Med. Chem.* **2015**, *58*, 9171–9178.

9. Q. Ge, Y. Hu, B. Li, B. Wang, *Org. Lett.* **2016**, *18*, 2483–2486.

10. R. Robiette, E. Van Den Berge, *J. Org. Chem.* **2013**, *78*, 12220–12223.

11. R. W. Cowgill, *J. Am. Chem. Soc.* **1957**, *79*, 2249–2251.

12. J. B. Lan, L. Chen, Q. X. Yu, J. S. You, R. G. Xie, *Chem. Commun.* **2004**, 188–189.

13. D. Liang, Y. Li, S. Gao, R. Li, X. Li, B. Wanga, H. Yang, *Green Chem.* **2017**, *19*, 3344–3349.

14. G.-F. Zha, W.-Y. Fang, J. Leng, H.-L. Qina, *Adv. Synth. Catal.* **2019**, *361*, 2262–2267.

15. P. E. Maligres, S. W. Krska, P. G. Dormer, *J. Org. Chem.* **2012**, *77*, 7646–7651.

16. CrystalClear: Rigaku Corporation, CrystalClear Software User's Guide, Molecular Structure Corporation, *Rigaku Corporation*, **1999**, 1718–1725.

17. G. M. Sheldrick, *Acta Cryst.* **2015**, *A71*, 3–8.

18. G. M. Sheldrick, *Acta Cryst.* **2015**, *C71*, 3–8 SHELXL Version 2018/3.

18. O. V. Dolomanov, L. J. Bourhis, R. J. Gildea, J. A. K. Howard, H. Puschmann, *J. Appl. Cryst.* **2009**, *42*, 339–341.

20. C. F. Macrae, P. R. Edgington, P. McCabe, E. Pidcock, G. P. Shields, R. Taylor, M. Towler, J. van de Streek, *J. Appl. Cryst.* **2006**, *39*, 453–457.

21. Persistence of Vision Pty. Ltd. (2004), Persistence of Vision Raytracer (Version 3.6).

22. S. Parsons, H. D. Flack and T. Wagner, *Acta Cryst.* **2013**, *B69*, 249–259.
